# Low-Temperature and High-Pressure Phase Transitions in Two 2‑Amino-4′-halobenzophenones: Incommensurate Modulation and a Case of Temperature-Induced Twinning

**DOI:** 10.1021/acs.cgd.5c01520

**Published:** 2026-02-06

**Authors:** Lani Attiwell, Max T. Hill, Jonathan D. Sellars, Lukáš Palatinus, Alexandra Longcake, Paul G. Waddell

**Affiliations:** † School of Natural and Environmental Sciences, Bedson Building, 5994 Newcastle University, Newcastle upon Tyne NE1 7RU, U.K.; ‡ Biosciences Institute, Faculty of Medical Sciences, Newcastle University, Newcastle upon Tyne NE1 7RU, U.K.; § School of Pharmacy, Faculty of Medical Sciences, Newcastle University, Newcastle upon Tyne NE1 7RU, U.K.; ∥ Department of Structure Analysis, Institute of Physics of the Czech Academy of Sciences, Na Slovance 1999/2, Prague 8 18221, Czechia

## Abstract

The structures of 2-amino-4′-chlorobenzophenone and 2-amino-4′-bromobenzophenone, previously determined at room temperature in the space group *Pna*2_1_, have been redetermined at low-temperature revealing two different reversible phase transitions. Additionally, high-pressure X-ray diffraction studies were conducted to allow for a comparison of the behavior of these structures in response to different external stimuli. Variable temperature analyses reveal that 2-amino-4′-bromobenzophenone transitions to the monoclinic space group *Pa* accompanied by a nonmerohedral twinning. The monoclinic phase exhibits approximate symmetry mimicking that of the supergroup *Pna*2_1_ but with a metric symmetry that precludes true *Pna*2_1_ symmetry. The structure of 2-amino-4′-chlorobenzophenone transitions to an incommensurately modulated phase upon cooling. The changes in the structure are attributed to slight conformational variations and a rearrangement of the hydrogen bonding networks in the structure in response to stimuli. The phase transition for 2-amino-4′-bromobenzophenone is classified as second order and a mechanism involving multiple coincident nucleation events is inferred from the conformational change and the incidence of twinning. The transformation of 2-amino-4′-chlorobenzophenone to an incommensurate phase is proposed to arise due to thermal contraction of the structure, which allows distinct competing hydrogen bonding networks between neighboring molecules to evolve.

## Graphical abstract

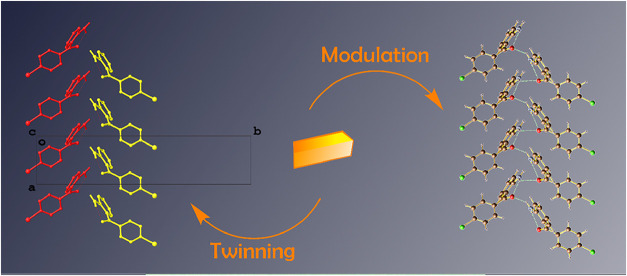

## Introduction

Benzophenones and their substituted derivatives are ubiquitous photophores with a long history of applications in photochemistry and medicinal chemistry.^1–3^ The benzophenone scaffold has been studied extensively in the solid-state with over 4000 structures containing this fragment returned by a search of the *Cambridge Structural Database* (CSD, version 5.45, update 2, Jun 2024). Crystallographic studies of the structures of substituted benzophenones have tended to concentrate on the conformational variation in the molecule^4,5^ and some efforts have been made to harness them in photocrystallographic experiments.^6,7^

Two examples of structures of substituted benzophenones are 2-amino-4′-bromobenzophenone and 2-amino-4′-bromobenzophenone (Figure 1), for which crystal structure data were reported in 1967 ^8^ and deposited with the *Cambridge Structural Database* in 1971 (CSD Refcode: AMBBPO and AMCBPO). These structures were observed to be isostructural and determined in the space group *Pna*2_1_ with one molecule in the asymmetric unit (*Z*′ = 1). During a study aiming to synthesize novel antibacterial agents, the structure of 2-amino-4′-bromobenzophenone was redetermined at 150 K revealing a new low-temperature polymorph, which is the subject of this study.

**Figure 1:**
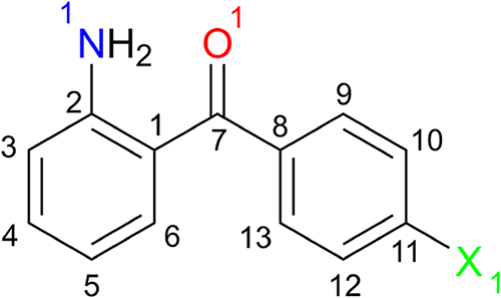
2-Amino-4′-halobenzophenone (X = Cl or Br) with the numbering scheme used in this article.

Upon further investigation, the crystals were found to undergo a completely reversible single-crystal-to-single-crystal (SCSC) phase transition between the known high-temperature form and the new low-temperature form, which has *Pa* space group symmetry and two molecules in the asymmetric unit (*Z*′ = 2). The SCSC is accompanied by a nonmerohedral crystal twinning. Such transitions involving twinning are known to occur in organic crystals but are rare.^9,10^

As the structure of the chloro analogue, AMCBPO, is isostructural with AMBBPO, 2-Amino-4′-chlorobenzophenone was also reinvestigated. A phase transition was observed at around 150 K for this compound, but in this case the low-temperature phase was incommensurately modulated. With our curiosity piqued, further measurements for both compounds were made at high-pressure, revealing incommensurately modulated phases similar to the low-temperature form of 2-Amino-4′-chlorobenzophenone in each.

This work investigates the nature of the phase transitions observed at low-temperature and at high-pressure in terms of the physical changes to the structures and the potential mechanisms behind them. In addition, this study provides a timely update to the original data for AMBBPO and AMCBPO with a more accurate and precise structure determination using modern methods and instrumentation.

## Experimental Section

2-Amino-4′-bromobenzophenone was purchased from Aldrich and 2-amino-4′-chlorobenzophenone from Fluorochem and were used without further purification. 2-Amino-4′-chlorobenzophenone was supplied in a form suitable for single crystal X-ray diffraction and crystals of 2-amino-4′-bromobenzophenone were grown as a mixture of yellow plates and yellow triangular prisms from a solution of the compound in dimethylformamide and water.

### Data Collection

Ambient pressure single crystal diffraction data were collected on a Rigaku XtaLAB Synergy-S diffractometer equipped with a HyPix-Arc 100 detector using copper radiation (λ_CuKα_ = 1.54184 Å) at temperatures between 140 and 290 K using an Oxford Cryosystems CryostreamPlus open-flow N_2_ cooling device. For consistency, one crystal of each compound was used for all analyses.

For 2-amino-4′-bromobenzophenone the sample was initially flash-frozen to 150 K and a full data set collected. Unit cell parameters were then measured at 5 K intervals heating to 190 K with a ramp rate of 120 K/h. A second full data set was collected at 190 K before the process was repeated in reverse, with further unit cell measurements made at 5 K intervals cooling to 150 K with a ramp rate of 120 K/h at which point the data collection at 150 K was repeated.

For 2-amino-4′-chlorobenzophenone a full data set was collected at 290 K before the sample was cooled to 140 K with a ramp rate of 120 K/h during which unit cell parameters were measured at 10 K intervals. A full data set was collected at 190 K to provide a direct comparison to the data for 2-amino-4′-bromobenzophenone collected at the same temperature. A data set corresponding to the incommensurately modulated phase was collected at 140 K.

Exposure times for the full collections were kept consistent; 0.5 s at low angle and 2 s at high-angle. Similarly, all unit cell measurements were made with 1 s exposures at low angle and 4 s exposures at high-angle. All measurements were made with a detector distance of 60 mm to improve the separation of the reflections corresponding to the two twin domains or satellite peaks.

High-pressure data and the associated reference collections at ambient pressure for 2-amino-4′-bromobenzophenone and 2-amino-4′-chlorobenzophenone were collected at 293(2) K on a Rigaku XtaLAB Synergy-S diffractometer equipped with a HyPix-Arc 100 detector and an Oxford Cryosystems open-flow N_2_ cooling device. The high-pressure and reference model data sets were collected using molybdenum (λ_MoKα_ = 0.71073 Å) and copper (λ_CuKα_ = 1.54184 Å) radiation, respectively.

The sample chambers of the custom built two screw Merrill-Bassett diamond anvil cells (DAC) used were formed by two 800 μm culet faces of Boehler-Almax cut diamonds, fitted into tungsten carbide backing seats. Stamped steel sheets (thickness 250 μm) were indented to a thickness of approximately 130 μm to form the gaskets. The gasket holes were drilled using a 380 μm diameter electrode on a BETSA electric discharge machine. Each sample was fixed to one culet face by means of high-vacuum hydrocarbon grease alongside two ruby spheres, which allowed for pressure measurement using the ruby fluorescence method.^11^ After each pressure ramp, the pressure inside the DAC was allowed to equilibrate for a minimum of 24 h before data collection was initiated. Pressure measurements were taken immediately before and after each collection and the pressure reported as the average. The error attributed to the inherent uncertainty of the ruby fluorescence method is approximately 0.05 GPa.^12^

A crystal of 2-amino-4′-bromobenzophenone was studied in Daphne-7373 and compressed to pressures of 0.0, 0.36, 0.69, 1.21, and 1.70 GPa. During decompression, further data sets were collected at 0.60 and 0.08 GPa. A crystal of 2-amino-4′-chlorobenzophenone was studied in Daphne-7373 and compressed to pressures of 0.0, 0.16, 0.66, 1.10, and 1.62 GPa. During decompression, further data sets were collected at 0.47 and 0.25 GPa. The hydrostatic limit of Daphne-7373 is reported to be approximately 2.2 GPa.^13^

### Data Processing

Cell refinement, data collection and data reduction were undertaken via the software CrysAlisPro.^14^ For data collected at high-pressure, special settings (DAC opening angle, data set resolution limits, profile rejection parameters and regular background updates) were implemented in the data reduction step to mitigate contamination of the data from diamond reflections and powder rings. The specific settings used are detailed in the CIF files for these structures.

For ambient pressure data, the intensities were corrected for absorption using a multifaceted crystal model created by indexing the faces of the crystal for which data were collected.^15^ For the high-pressure data, spherical absorption corrections were applied along with an empirical absorption correction using spherical harmonics, as implemented in the SCALE3 ABSPACK scaling algorithm within CrysAlisPro.^14^

All structures were solved using XT^16^ with the exception of the data collected at high-pressure. In this case, initial collections conducted outside of the DAC at ambient pressure for each structure were solved using XT and subsequent high-pressure data sets conducted inside the DAC were solved by importing a reference model from either the ambient condition collection or an appropriate model from the previous lower pressure point. All structures were refined by XL^17^ using the Olex2 interface.^18^ The space group *Pa* was chosen for the low-temperature phase of 2-amino-4′-bromobenzophenone to preserve the orientation of the axes in the high-temperature phase.

For the ambient pressure data, all non-hydrogen atoms were refined using anisotropic displacement parameters (ADPs) and hydrogen atoms were positioned with idealized geometry, with the exception of those bound to heteroatoms, the positions of which were located using peaks in the Fourier difference map. The displacement parameters of the hydrogen atoms were constrained using a riding model with *U*_iso_ set to be an appropriate multiple of the *U*_eq_ value of the parent atom. For all high-pressure data sets, in order to preserve the data-to-parameter ratio as much as possible, all non-hydrogen atoms were refined using isotropic displacement parameters, with the exception of the halogen atoms, which were refined using anisotropic displacement parameters, where possible. For the high pressure data sets of 2-amino-4′-chlorobenzophenone collected at 0.47, 0.66, 1.10, and 1.62 GPa, where the structure exhibits incommensurate modulation and an average structure is reported, the chlorine atoms are also refined using isotropic displacement parameters. Specific refinement details are detailed in the CIF files for these structures.

As structural solutions that accounted for the modulation were not obtained for any of the high-pressure data sets, the reduced data (in which the satellite peaks are integrated) and selected unwarped frames are supplied alongside the Supporting Information, in addition to CIFs in which the average structures are reported (where data reduction was carried out on only the main reflections).

The incommensurately modulated phase of 2-amino-4′-chlorobenzophenone at 140 K and ambient pressure was solved directly in superspace by the program Superflip^19,20^ within Jana2020.^21^ This method provides a superspace electron density, which allows an *ab initio* determination of both the average structure and its modulation. The structure was solved successfully, and the complete 2-amino-4′-chlorobenzophenone molecule could be located in the solution. The refined q-vector has a nonzero component close to 1/6, such that the third-order satellite reflections almost exactly overlapped with the third-order satellites of the neighboring reflections. As a result, the refinement was conducted taking the fact that the third-order satellites are overlapping into account. The modulation was described using harmonic modulation waves up to the third order for atomic positions and ADPs for all non-hydrogen atoms. The hydrogen atoms were treated as riding, fixed in geometrically predicted positions.

## Results and Discussion

### Diffraction

The low-temperature, twinned, monoclinic phase of 2-amino-4′-bromobenzophenone was discovered serendipitously as standard experiments in our laboratory are performed at 150 K and this phase was induced when the crystal was flash-frozen (Table 1). The structure was initially thought to be a redetermination of AMBBPO as both exhibit very similar unit cell parameters and crystal packing. If this were the case the space group of the structure of AMBBPO would have to have been misassigned. This was considered a possibility as the earlier data were collected on photographic film and analyzed visually, a procedure much less precise and more prone to error than modern computer-based methods. It seemed possible that the β angle, being close to 90°, could have been rounded down to give the orthorhombic metric symmetry reported. It also seemed possible that, if the same twinning was observed in the diffraction pattern of AMBBPO, that this may have further clouded the issue.

**Table 1:** Crystal Data and Structural Refinement Details for 2-Amino-4′-bromobenzophenone

	150 K	190 K
empirical formula	C_13_H_10_BrNO	
formula weight	276.13	
crystal system	monoclinic	orthorhombic
space group	*Pa*	*Pna*2_1_
*a*/Å	7.67458(10)	7.7727(2)
*b*/Å	25.5980(3)	25.4003(8)
*c*/Å	5.76382(8)	5.7980(2)
α/°	90	90
β/°	92.6639(11)	90
γ/°	90	90
volume/Å^3^	1131.10(2)	1144.69(6)
*Z*	4	4
ρ_calc_g/cm^3^	1.622	1.602
μ/mm^–1^	4.757	4.701
*F*(000)	552.0	552.0
crystal size/mm^3^	0.19 × 0.07 × 0.02	0.18 × 0.07 × 0.02
radiation	Cu Kα (λ = 1.54184)	Cu Kα (λ = 1.54184)
2Θ range for data collection/°	6.906 to 146.72	6.96 to 146.61
index ranges	–9 ≤ *h* ≤ 9, –31 ≤ *k* ≤ 31, –7 ≤ *l* ≤ 5	–9 ≤ *h* ≤ 9, –28 ≤ *k* ≤ 30, –4 ≤ *l* ≤ 6
reflections collected	20047	4758
independent reflections	2246 [*R*_int_ = 0.0263, *R*_sigma_ = 0.0202]	1612 [*R*_int_ = 0.0159, *R*_sigma_ = 0.0184]
data/restraints/parameters	20047/2/302	1612/1/151
goodness-of-fit on *F* ^2^	1.051	1.068
final R indexes [*I* ≥ 2σ (*I*)]	*R*_1_ = 0.0250, w*R*_2_ = 0.0677	*R*_1_ = 0.0194, w*R*_2_ = 0.0539
final R indexes [all data]	*R*_1_ = 0.0255, w*R*_2_ = 0.0681	*R*_1_ = 0.0198, w*R*_2_ = 0.0543
largest diff. peak/hole/e Å^–3^	0.30/–0.22	0.23/–0.25
flack parameter	–0.021(11)	–0.02(2)

As AMBBPO was collected at room temperature, the unit cell parameters of the crystal were measured at 290 K in order to replicate the conditions of the original experiment. At this temperature the diffraction pattern exhibited no signs of twinning and was indexed with an orthorhombic unit cell and similar unit cell parameters to AMBBPO, confirming that this determination was accurate and there was indeed a distinct low-temperature phase at 150 K. Full data collected at 290 K are available in the Supporting Information.

To probe the nature of the phase transition, a variable temperature experiment was proposed. Attempts to cool a crystal slowly from the orthorhombic to the monoclinic phase were not successful as the crystals for which data were collected in this manner did not diffract well after the phase transition, exhibiting streaky, arced reflection profiles, though the transition was seen to be reversible upon returning to a higher temperature. It would seem that a crystal robust enough to endure the phase transition was required but as it is uneconomical in terms of time and resources to start collecting data at high-temperature and cool the sample until the phase transition, only then to discover that the diffraction was poor, another approach was required.

As good quality data had already been collected for a flash-cooled crystal and the transition was observed to be reversible, the decision was made to flash-cool a crystal to 150 K and then raise the temperature until the orthorhombic phase was observed. In this way, after trialling a number of crystals to find one for which all observed reflections could be indexed as a two-component twin, the quality of the diffraction at low-temperature could be guaranteed before the start of the variable temperature experiment.

Once a suitable crystal (one that produced strong diffraction and for which all observed reflections could be indexed as a two-component twin at 150 K) was identified, a data set was collected for the low-temperature twinned phase. The temperature was then increased with unit cell parameters being measured at 5 K intervals. The β angle was observed to decrease from 92.601(2)° at 150 K to 90.34(18)° at 180 K as the structure approached the transition to the orthorhombic phase. At 185 K, the reflections could be indexed as the orthorhombic phase, though very slight splitting of a few reflections was still observed. At 190 K, the diffraction pattern exhibited no split reflections and a data set corresponding to the high-temperature orthorhombic phase was collected (Figure 2).

**Figure 2:**
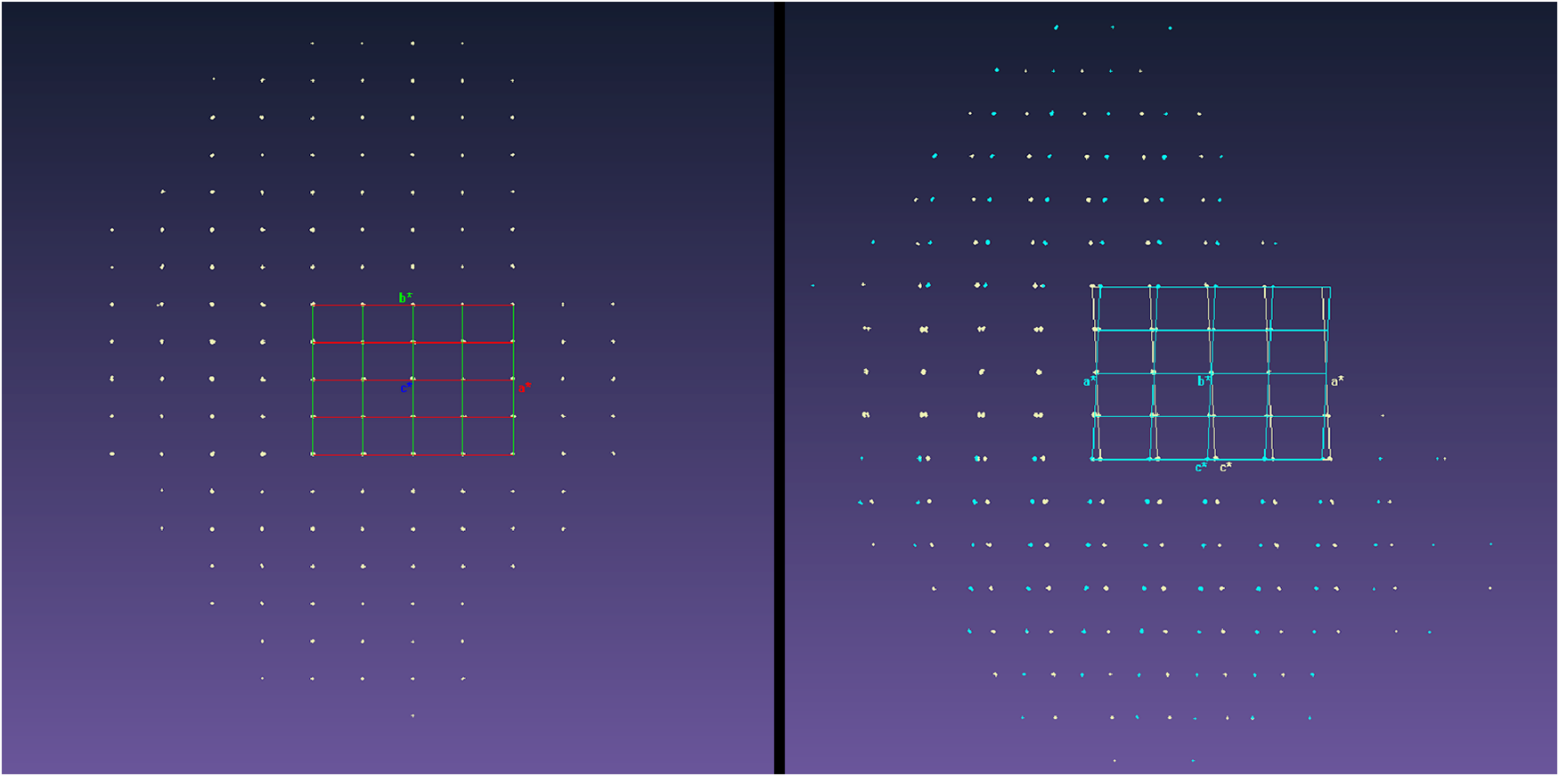
Reciprocal lattices observed for crystals of 2-amino-4′-bromobenzophenone at 190 (left) and 150 K (right) viewed down the equivalent c* and b* reciprocal lattice vectors, respectively.

Unit cell parameters were then measured at 5 K intervals descending in temperature to 150 K. The first instances of split reflections were observed at 180 K though the crystal still seemed to be predominantly the orthorhombic phase. At 175 K the reflections could be indexed as a two-component twin again and the β angle was measured to be 91.07(15)°. The difference in the observed phase transition temperatures between the heating and cooling modes suggests a slight hysteresis between the two.

Further evidence of a hysteresis is observed in the unit cell parameters (Figure 3). Where the *a*-axis follows a more linear trend with only a suggestion of hysteresis upon cooling, the *b*- and *c*-axes show a more noticeable hysteresis. As there is little variation in the *c*-axis compared to the *b*-axis, and the errors appear more significant for the <6 Å distance, the trend in the latter is probably more reliable in terms of assessing this hysteresis. The profile for the unit cell volume resembles that of the *c*-axis and is available along with that of the *a*-axis in the Supporting Information.

**Figure 3:**
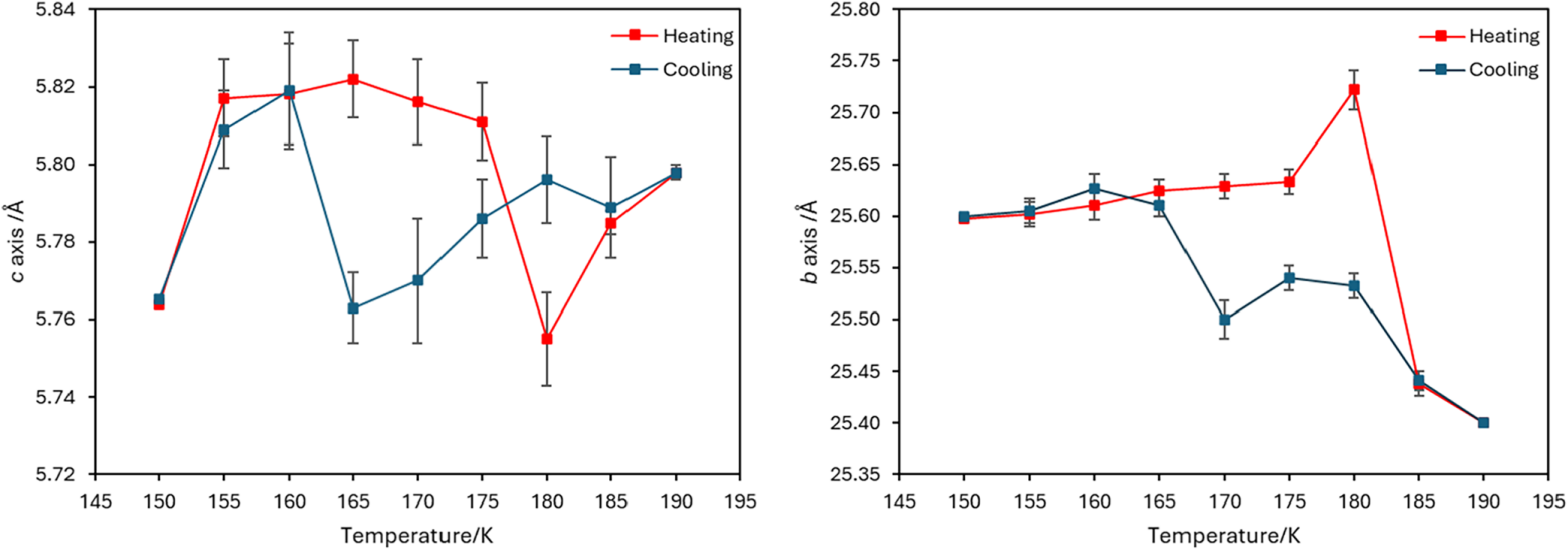
Temperature profiles of selected axes for 2-amino-4′-bromobenzophenone during heating and cooling.

Upon heating, after a sharp expansion in the *c*-axis between 150 and 155 K, both *b*- and *c*-axes remain relatively stable until 180 K. At this point there is a simultaneous contraction of the *c*-axis and an expansion of the *b*-axis as the crystal approaches the phase transition. At 185 K, where the orthorhombic form is first observed, the *b*-axis contracts to a length below that observed at 150 K and the *c*-axis expands signaling the completion of the phase transition. Upon cooling, the *b*-axis undergoes a slight expansion before shortening after 175 K where the transition to the twinned monoclinic phase is observed. By 160 K, the profiles of the cooling regimes coalesce.

At 150 K, a second data set for the low-temperature phase was measured. The structure determination was of equivalent quality to the first data set collected at 150 K demonstrating that the phase transition was fully reversible (enantiotropic). Full data for this structure are available in the Supporting Information.

For 2-amino-4′-chlorobenzophenone, as the structure was known to be isostructural with 2-amino-4′-bromobenzophenone, a phase transition was anticipated and hence a good quality crystal was mounted at 290 K and unit cell measurements made at 10 K intervals until a phase transition was observed. Full data sets were collected at 290 K (available in the Supporting Information) and 190 K (Table 2) to provide a direct comparison to those of 2-amino-4′-bromobenzophenone collected at the same temperatures.

**Table 2:** Crystal Data and Structural Refinement Details for 2-Amino-4′-chlorobenzophenone

	140 K^a^	190 K
empirical formula	C_13_H_10_ClNO	
formula weight	231.67	
crystal system	orthorhombic	
space group	*Pna*2_1_	
*a*/Å	7.6489(4)	7.8190(2)
*b*/Å	25.2922(14)	25.0585(8)
*c*/Å	5.6733(4)	5.6629(2)
α/°	90	90
β/°	90	90
γ/°	90	90
volume/Å^3^	1097.54(11)	1109.55(6)
*Z*	4	4
ρ_calc_g/cm^3^	1.402	1.387
μ/mm^–1^	2.876	2.845
*F*(000)	480.0	480.0
crystal size/mm^3^	0.18 × 0.07 × 0.02	0.17 × 0.07 × 0.02
radiation	Cu Kα (λ = 1.54184)	Cu Kα (λ = 1.54184)
2Θ range for data collection/°	6.99 to 147.06	7.056 to 146.882
index ranges	–9 ≤ *h* ≤ 9, –31 ≤ *k* ≤ 30, –4 ≤ *l* ≤ 7	–9 ≤ *h* ≤ 9, –30 ≤ *k* ≤ 31, –7 ≤ *l* ≤ 4
reflections collected	9121	9886
independent reflections	1590 [*R*_int_ = 0.0238, *R*_sigma_ = 0.0137]	1568 [*R*_int_ = 0.0213, *R*_sigma_ = 0.0148]
data/restraints/parameters	1590/1/151	1568/1/151
Goodness-of-fit on *F* ^2^	1.109	1.083
final R indexes [*I* ≥ 2σ (*I*)]	*R*_1_ = 0.0629, w*R*_2_ = 0.1435	*R*_1_ = 0.0243, w*R*_2_ = 0.0686
final R indexes [all data]	*R*_1_ = 0.0641, w*R*_2_ = 0.1444	*R*_1_ = 0.0250, w*R*_2_ = 0.0694
largest diff. peak/hole/e Å^–3^	0.36/–0.40	0.11/–0.15
flack parameter	0.04(5)	0.000(9)

a Average structure.

At 150 K, the diffraction pattern began to exhibit signs of a phase transition. Weak satellite peaks consistent with incommensurate modulation were observed. At this temperature these satellite peaks seemed a little too close together to resolve so the decision was made to cool further to 140 K to ensure a full transition. The satellite peaks appeared clearer at this temperature and a full data set was collected. As with the transition observed in 2-amino-4′-bromobenzophenone, the transition to this incommensurate phase was observed to be fully reversible.

In terms of the temperature profiles of 2-amino-4′-chlorobenzophenone, a decrease in all axes and the unit cell volume was observed from 290 K up until the phase transition but the most significant contraction was observed for the *c*-axis. In contrast the overall contraction of the *a*- and *b*-axes over this temperature range were very slight (Figure 4), with the *a*-axis in particular almost appearing to plateau. As a result, the trend in the unit cell volume matches the profile of that of the *c*-axis both of which are available in the Supporting Information.

**Figure 4:**
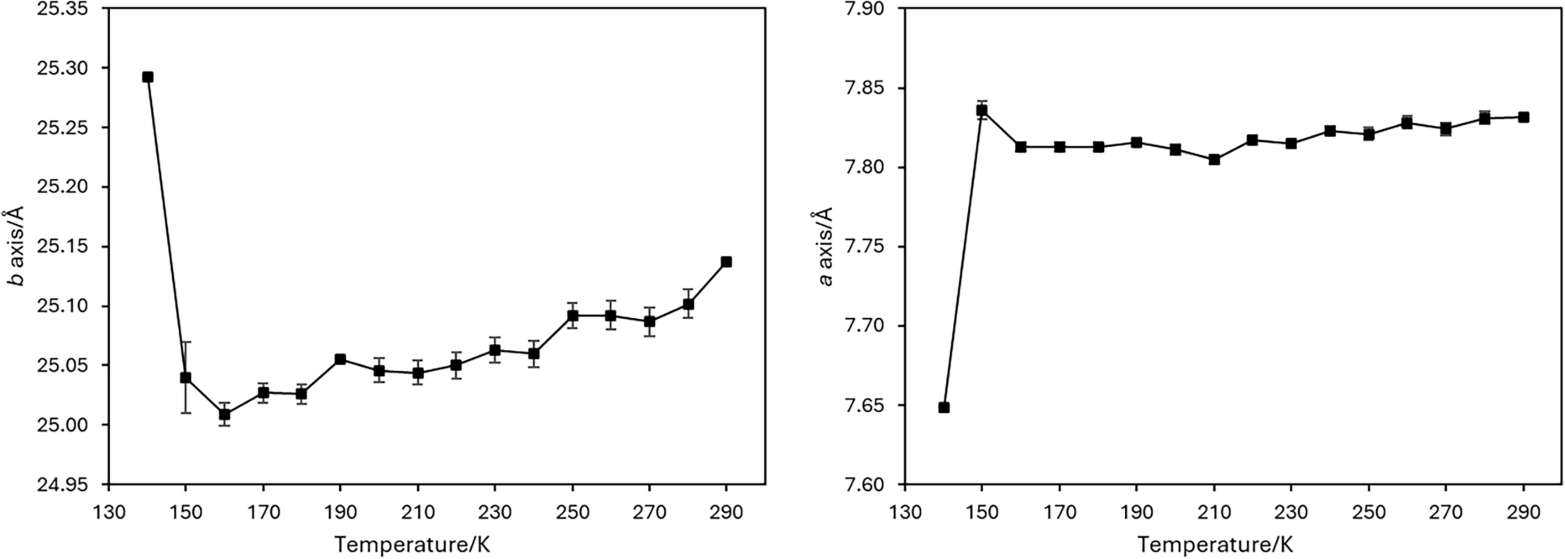
Temperature profiles of selected axes for 2-amino-4′-chlorobenzophenone during cooling.

At the point of the transition, the unit cell volume increases slightly at 150 K before contacting at 140 K to a value that fits the overall trend in the volume upon cooling. Significant deviations in the temperature profiles of all the axes and observed at 140 K. The *a*-axis contracts by over 0.15 Å between 150 and 140 K, more than twice as much as it contracted between 290 and 160 K. This contraction is accompanied by sharp expansions along the b- and c-axes suggesting a significant rearrangement. For the *b*-axis this expansion is particularly pronounced.

These trends suggest that upon cooling, as the *a*- and *b*- axes decrease only slightly before the phase transition, that there is significant strain building in these directions up until the point where the structure must rearrange significantly to relieve the building pressure and it is this that leads to the transition to the incommensurately modulated phase.

In terms of the diffraction pattern, sets of satellite reflections indicative of modulation are clearly resolved at 140 K (Figure 5). A complete description of the structure, therefore, requires the determination of this modulation.^22^ The modulation vector refined to *q* = (0.16255(11), 0, 0), which is very close to the commensurate value of 0.1667a* yet sufficiently different that the modulation can confidently be ascribed as being incommensurate, with a 6-fold superstructure being a reasonable commensurate approximation of the structure. Since this low-temperature data set was collected to high-resolution and with close to 100% completeness a description of the structure in (3 + 1)-dimensional superspace proved feasible.

**Figure 5:**
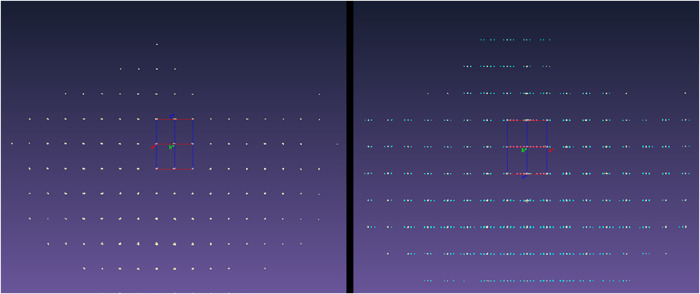
Reciprocal lattices observed for crystals of 2-amino-4′-chlorobenzophenone at 190 (left) and 140 K (right) viewed down the b* reciprocal lattice vectors. The main reflections and satellite reflections are depicted in pale yellow and cyan, respectively.

### Crystal Structures

Considering the crystal structure determinations for 2-amino-4′-bromobenzophenone at 150 and 190 K, the differences between the two are subtle but have a drastic effect on the crystal system and space group symmetry. At 190 K the structure exhibits orthorhombic *Pna*2_1_ symmetry with one molecule in the asymmetric unit (*Z* = 4, *Z*′ = 1) where at 150 K (Figure 6) the structure is monoclinic with *Pa* space group symmetry and two molecules in the asymmetric unit (*Z* = 4, *Z*′ = 2). As the unit cell parameters do not change significantly, with the exception of the β angle, which is still relatively close to 90° for the monoclinic phase, the salient difference between the two structures would seem to be that there are two molecules in the low-temperature structure that are not related by crystallographic symmetry. The relationship between these two molecules would presumably be the key to understanding the nature of the phase transition.

**Figure 6:**
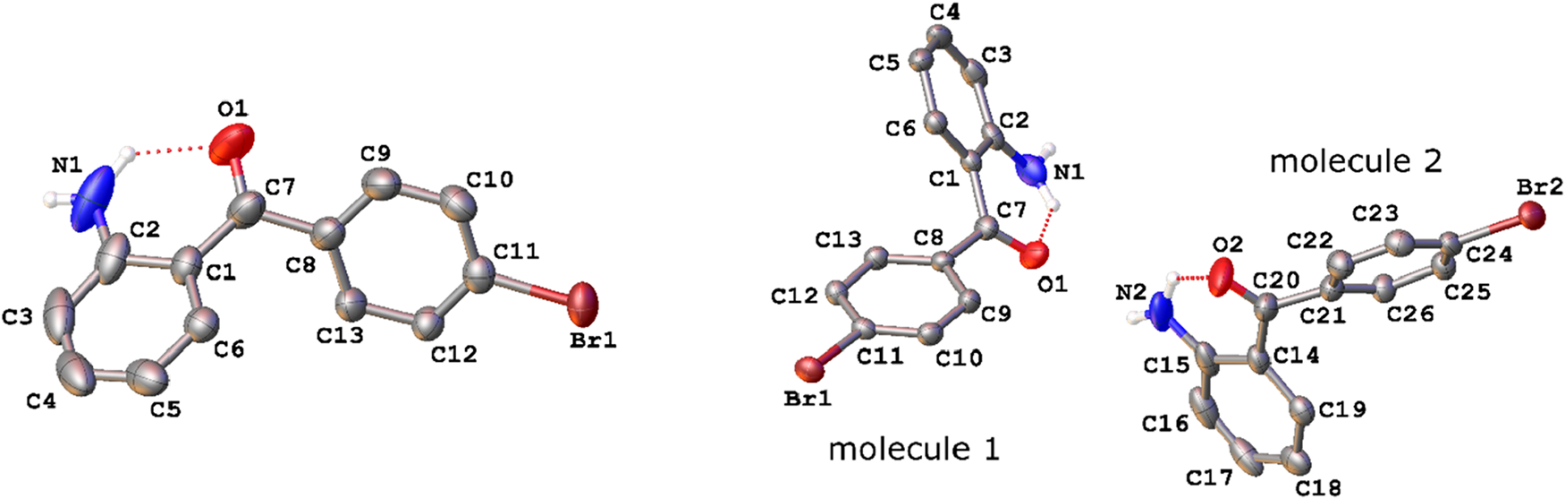
Asymmetric units of the structure of 2-amino-4′-bromobenzophenone at 190 K (left) and 150 K (right) with ellipsoids drawn at the 50% probability level. Hydrogen atoms bound to carbon atoms have been omitted for clarity.

In terms of molecular geometry, the bond distances of the two independent molecules in the asymmetric unit of the structure at 150 K do not vary significantly but the anisotropic displacement parameters (ADP) of molecule 2 are larger on average than those of molecule 1 and directed in a manner that suggests a degree of dynamic disorder in the form of libration. Overlaying the molecules reveals that there is a significant conformational variation between the two (Figure 7). This is best quantified by the C6–C1–C7–C8 torsion angle, which is *ca*. 28.0° for molecule 1 and *ca*. 33.5° for the equivalent torsion angle in molecule 2 (C19–C14–C20–C21). This is clearly the reason for the *Z*′ value of 2 observed for this structure.

**Figure 7:**
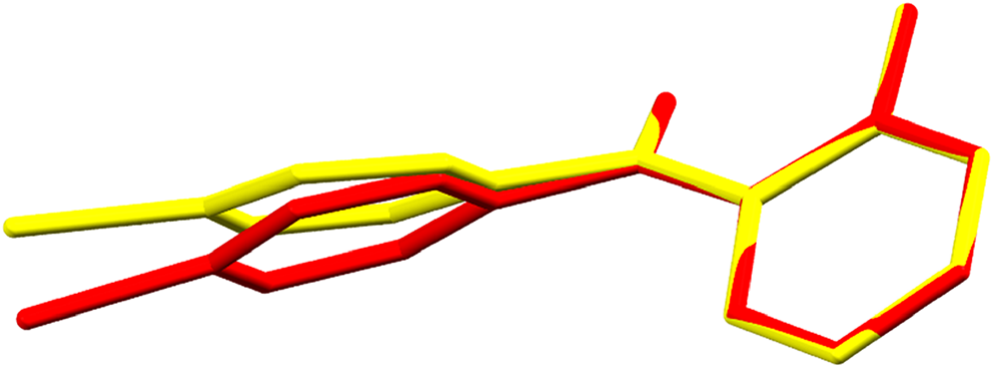
Overlay of molecule 1 (red) and molecule 2 (yellow) in the asymmetric unit of 2-amino-4′-bromobenzophenone at 150 K.

When compared to the structure at 190 K, the molecules at this temperature best match those of molecule 1 at 150 K as the two overlay almost exactly and there is no significant difference in the C6–C1–C7–C8 torsion angle, which is *ca*. 28.9° at 190 K. Given this, it can be concluded that only one in every pair of molecules in the structure at 190 K changes conformation through the phase transition.

Viewing the structure at 150 K along the [001] direction, it is apparent that the two independent molecules that comprise the asymmetric unit are related by an approximate screw axis. This corresponds to the real 2_1_ screw axis in the [001] direction in the structure at 190 K. At the lower temperature, the symmetry of the screw axis is clearly broken by the change in conformation observed in molecule 2 (Figure 8). This is also true of the *n*-glide in the [011̅] direction at 190 K, the vestiges of which are still manifest at 150 K as approximate glide symmetry. Again, the symmetry here is broken by the two different conformations in the asymmetric unit which are observed on either side of the approximate glide plane.

**Figure 8:**
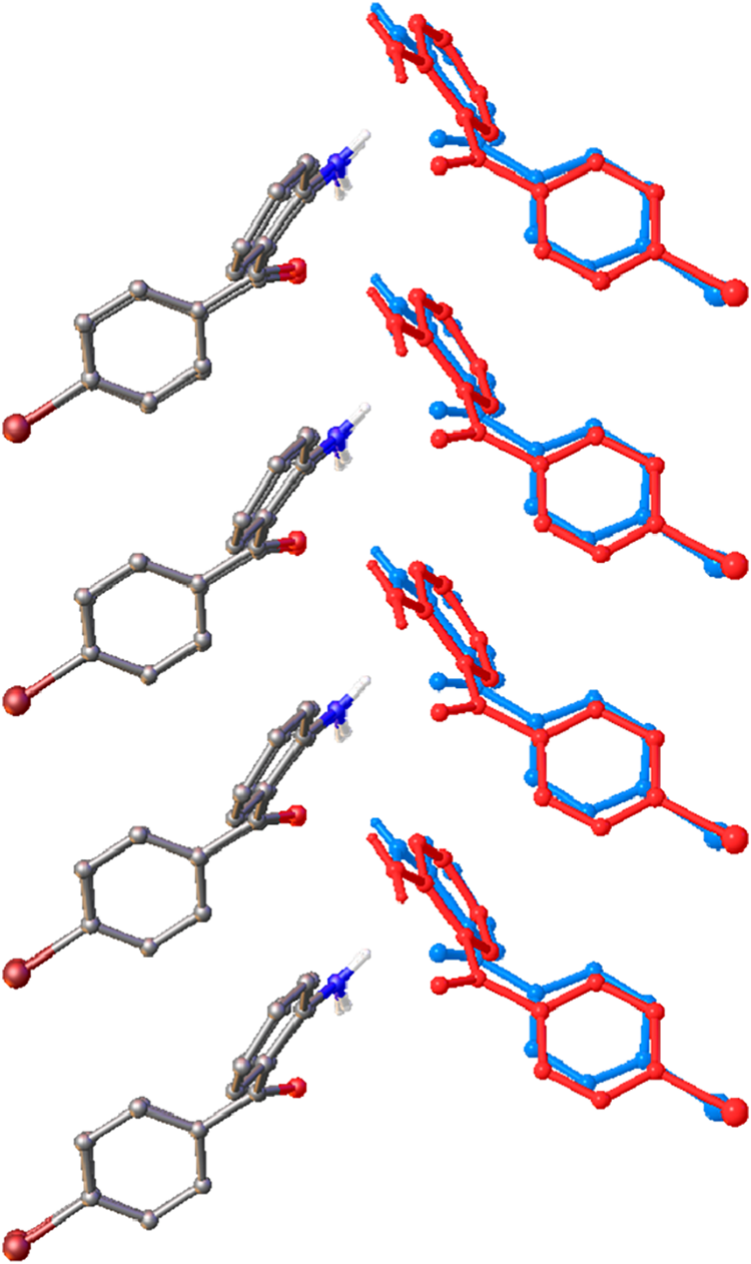
Overlay of the 2_1_ screw axis motif in the structure of 2-amino-4′-bromobenzophenone at 190 K with the approximate screw axis motif in the structure at 150 K. Symmetry equivalent molecules at 190 K (red) rearrange at 150 K (blue) and break the crystallographic symmetry.

The phase transition also leads to a subtle change in the hydrogen bonding motif observed in the direction of the screw axis at 190 K. At the lower temperature the hydrogen bonding interaction appears almost bifurcated. The precise geometry is unclear due to the libration of the nitrogen atom but as each molecule in the chain is symmetry equivalent the interaction must also be consistent along the chain. Regardless of which proton on the amine group is the donor atom, the chain can be described as a one-dimensional chain with a C(6) graph set.^23^ By way of contrast, at 150 K the rearrangement results in a chain of hydrogen bonds where the donor proton alternates between H1A, which is involved in an intramolecular bond, and H2B (Table 3). Though these interactions appear weak there is clearly one distance shorter than the other and a noticeable change in the orientation of the amine group relative to the carbonyl of each adjacent molecule.

**Table 3:** Hydrogen Bond Geometry for the Structure of 2-Amino-4′-bromobenzophenone at **150 K**

D–H···A	*d*(D–H)/Å	*d*(H···A)/Å	*d*(D···A)/Å	D–H···A/°
150 K				
N1–H1A···O2^a^	0.95(7)	2.41(7)	3.012(7)	121(5)
N2–H2B···O1	0.89(8)	2.53(9)	2.949(6)	112(7)

a +*X*, +*Y*, 1 + *Z*.

The breaking of the *Pna*2_1_ symmetry at 150 K is accompanied by a change in the β angle, which can also be attributed to the conformational change observed in molecule 2. This perturbation shifts the relative positions of neighboring equivalents of molecule 1 so that they are no longer periodic along what would be the [001] direction at 190 K but instead are offset in this direction by an angle greater than 90° (Figure 9). It is this change in the metric symmetry that precludes glide planes and screw axes in all but one direction and leads to the formation of the approximate symmetry observed at 150 K.

**Figure 9:**
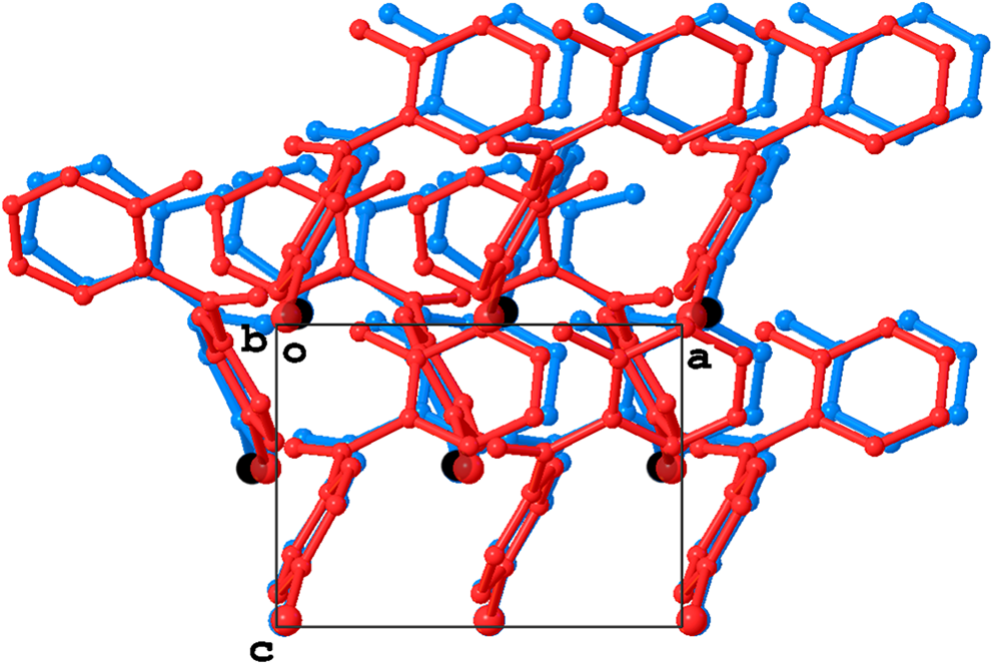
An overlay of the packing in the structure of 2-amino-4′-bromobenzophenone at 190 K (red) and 150 K (blue). Three bromine atoms of molecule 1 at 150 K have been overlaid directly with those at 190 K highlighting the change in packing brought about by the phase transition. The bromine atoms of the structure at 150 K that do not overlay with those at 290 K have been highlighted with black circles to show the effects of the conformational change in molecule 2 and the subtle change in the β angle as the structure transitions to monoclinic metric symmetry.

That a very slight molecular perturbation leads to a transition from *Pna*2_1_ to *Pa* is not entirely unexpected. *Pna*2_1_ is a supergroup of *Pa* and the structure at 150 K can be described as mimicking *Pna*2_1_ symmetry, retaining the 2_1_ screw axis and *n*-glide motifs in the form of approximate symmetry. There are a handful of examples of structures in *Pa* being related to high-symmetry phases in a similar fashion,^24^ and, though not explicitly proven, for the polymorphs of methyl 2-(9H-carbazol-9-yl)­benzoate, there is an example where a *Pa* phase (in the setting *Pn*) is almost certainly related to one in *Pna*2_1_.^25,26^

Though isostructural with 2-amino-4′-bromobenzophenone, to the point where they are practically identical at 190 K, the phase transition observed at low-temperature for 2-amino-4′-chlorobenzophenone is markedly different and this can be observed in the structure. The structure of 2-amino-4′-chlorobenzophenone at 140 K is incommensurately modulated and, hence, a description of the structure in (3 + 1)-dimensional superspace is necessary.

Consistent with the structure prior to the phase transition, the average structure at 140 K is orthorhombic, space group *Pna*2_1_; but when solved in superspace, the superspace group was determined to be *Pna*2_1_(α00)­000 by the analysis of the *ab initio* solution obtained by Superflip.^27^ The modulation is relatively strong with the modulation amplitudes approaching 1 Å for many atoms. Despite this strong modulation, the geometry of the molecule remains rigid with the majority of the modulation affecting the translations and rotation of the molecule as a whole. The most strongly modulated internal geometrical features are the two free torsions between the two aromatic rings of the benzophenone molecule (Figure 10) with a total modulation amplitude of approximately 9°, similar to the variation in conformation observed in 2-amino-4′-bromobenzophenone.

**Figure 10:**
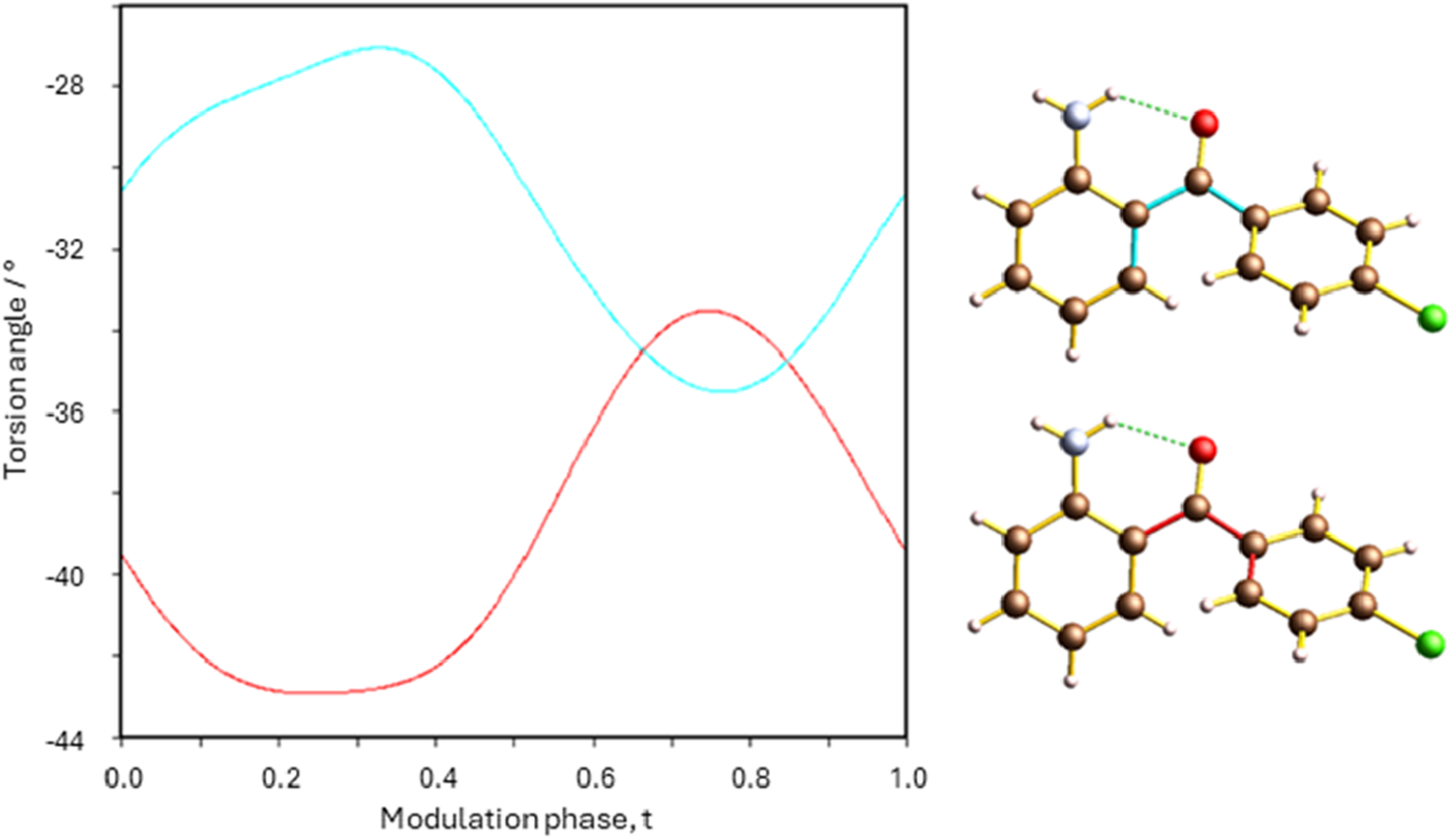
*t*-Plot of C6–C1–C7-C8 (cyan) and C1–C7–C8-C13 (red) torsion angles as a function of modulation phase. Insets on the right depict the two torsion angles plotted.

The origin of the modulation in this structure can be attributed to two separate competing hydrogen-bonding networks present in the modulated phase (Figure 11). For the purposes of the following discussion, a hydrogen bond is defined by donor–acceptor O···H distances of ≤2.5 Å and N–H···O bond angles of ≥110°.^28^ In addition to the ubiquitous intramolecular S(6) hydrogen bond observed in both 2-amino-4′-chlorobenzophenone and 2-amino-4′-bromobenzophenone, intermolecular interactions N1–H1···O1 and N1–H2···O1 between neighboring molecules are observed at different points in phase space. As observed in 2-amino-4′-bromobenzophenone, these interactions form chains of molecules along the [001] direction with a C(6) graph set.

**Figure 11:**
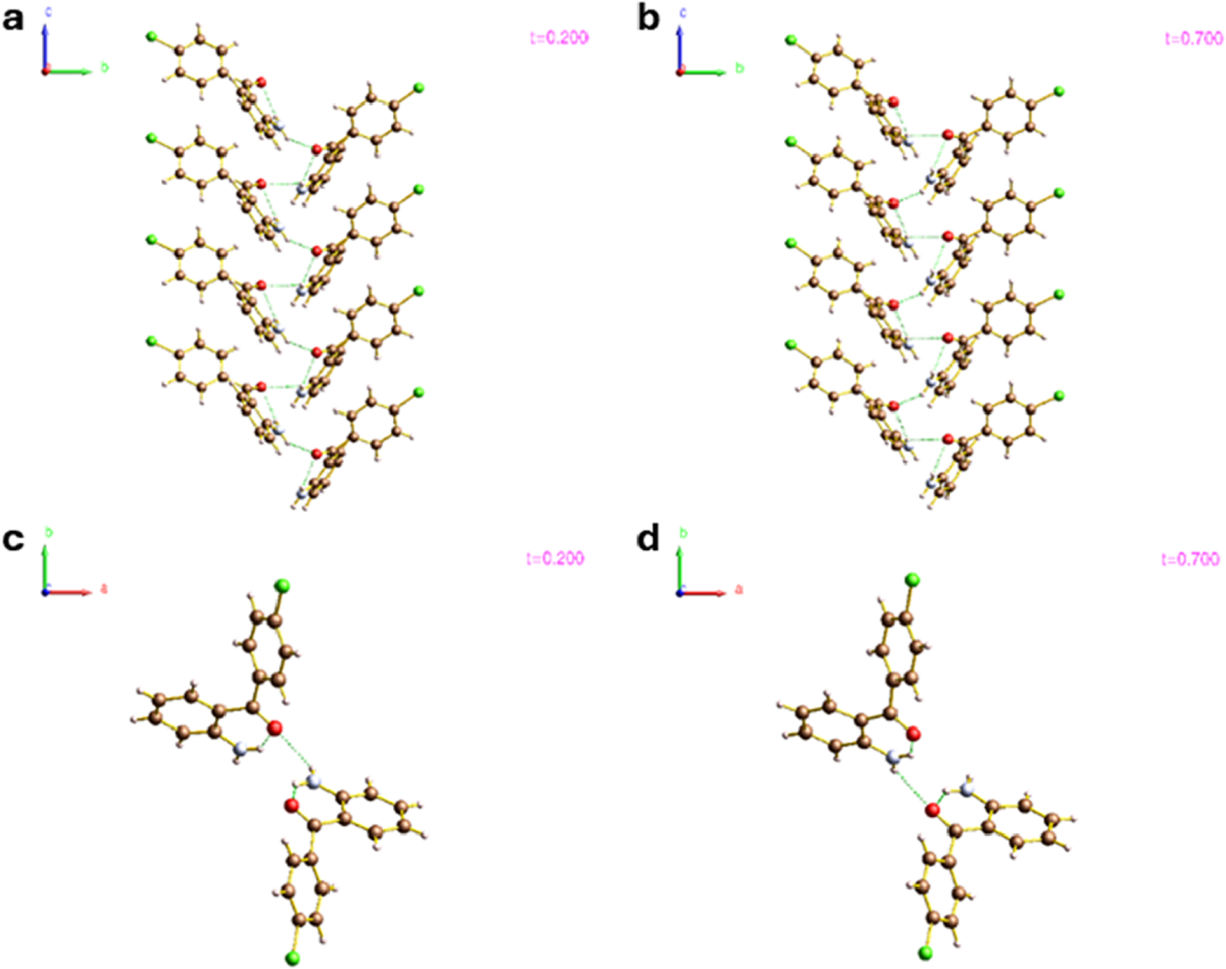
Ball and stick depiction of the two distinct hydrogen bonding networks competing within the structure of **2** at different modulation phases, as viewed down the [100] direction at *t* = 0.2 (a) and *t* = 0.7 (b). Depictions of the intermolecular N1–H2···O1 hydrogen bond between a pair of molecules at *t* = 0.2 (c) and *t* = 0.7 (d), as viewed down the *c*-axis, are also supplied. The N1–H1···O1 hydrogen bond is not shown in the bottom two figures, for clarity. The hydrogen bonds are depicted by green dotted lines.

As the phase of the modulation (*t*) evolves, the respective donor/acceptor atoms (N1–H1···O1 and N1–H2···O1) involved in these intermolecular hydrogen bonds alternate between the two molecules in the interacting pair. At *t* = 0.2 in Figure 11c, an intermolecular hydrogen bond is observed where the N1–H2 proton of the lower right molecule donates to the O1 atom of the neighboring top left molecule, as viewed down the [001] direction. This intermolecular interaction propagates the C(6) chain along the [001] direction. However, as the modulation phase evolves toward t = 0.7 (Figure 11d), the associated geometrical rearrangement brings the O1 atom of the lower right molecule into closer proximity of the N1–H2 atom, such that a second distinct intermolecular hydrogen bonding network forms that is also comprised of continuous N1–H1···O1 and N1–H2···O1 interactions, where the donor and acceptor molecules have essentially switched position (Figure 2b).

The formation of this second distinct hydrogen bonding network at *t* = 0.7 comes at the expense of the first hydrogen bonding network observed at *t* = 0.2. As a result, these two competing hydrogen-bonding networks within the structure give rise to the modulation observed at 140 K, because the coexistence of these two distinct hydrogen bonding networks is precluded geometrically. The N1–H1···O1 and N1–H2···O1 distances as a function of the modulation phase, *t*, show the interplay between the different hydrogen bonding networks within the modulated structure (Figure 12). The antiphase behavior of the N1–H1···O1 and N1–H2···O1 distances as a function of t again demonstrates the competition observed between the two distinct hydrogen bonding networks described above.

**Figure 12:**
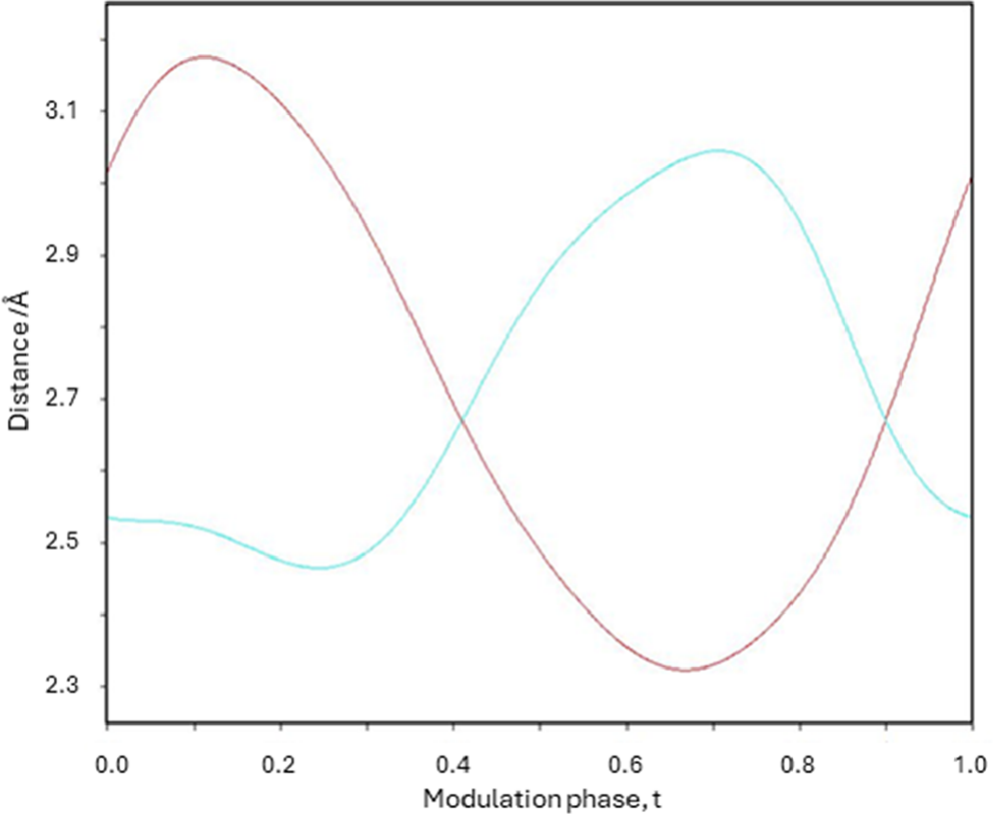
*t*-Plot of the N1–H1···O1 (cyan) and N1–H2···O1 (red) distances involved in the hydrogen bonding networks of 2-amino-4′-chlorobenzophenone as a function of modulation phase.

The modulation itself is characterized by a smooth sinusoidal shape with no noticeable discontinuities to the function, indicating that the transition between the two distinct hydrogen bonding networks is smooth and continuous, with no abrupt interruptions within the structure as a function of phase space. The de Wolff sections for N1 and O1 involved in the hydrogen bonding network are provided in Figure 13, and show the respective *x*1 – *x*4, *x*2 – *x*4 and *x*3 – *x*4 sections through superspace electron density.

**Figure 13:**
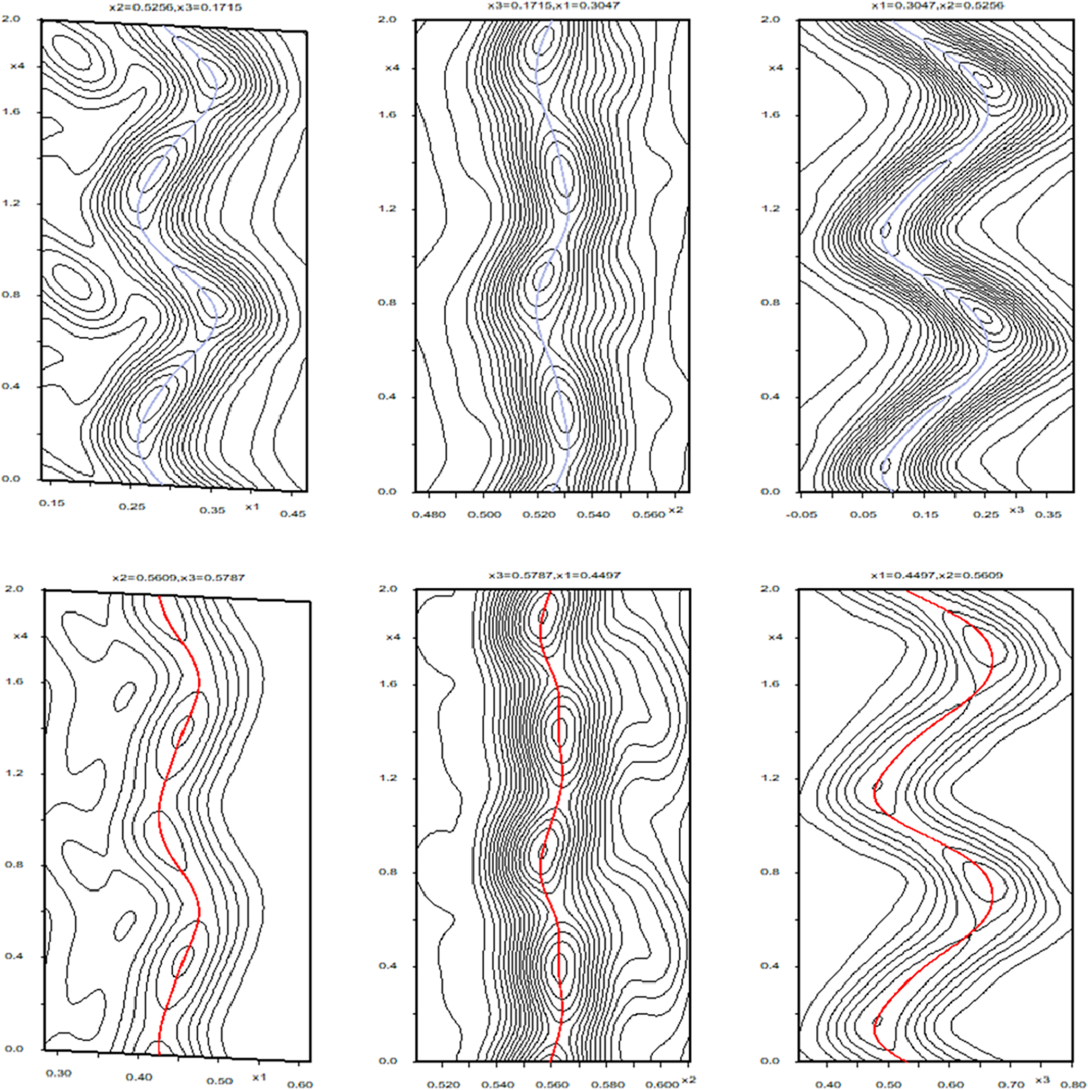
Summation Fourier maps in superspace of the *x*1 – *x*4, *x*2 – *x*4 and *x*3 – *x*4 sections (left to right) for N1 (top) and O1 (bottom). The respective solid blue and red lines running through the middle of each plot indicate the calculated position of N1 and O1 at that point in phase space. For clarity, the maps contain two modulation periods.

An approximate model of the true modulated structure can be constructed using a 6-fold commensurate superstructure in the space group *Pn*. This approximation can be obtained from the incommensurate structure by adjusting the modulation vector to the commensurate value of 1/6a* via a mathematical transformation within Jana2020. The resulting supercell (Figure 14) depicts the variation of the hydrogen bonding network across every six pairs of atoms and is a reasonable periodic approximation of the true incommensurate structure. Note how two distinct hydrogen bonding motifs are clearly visible within the supercell. Additionally, it should be acknowledged that this is merely an approximate representation of the true modulated structure, which requires a superspace description to be truly meaningful.

**Figure 14:**
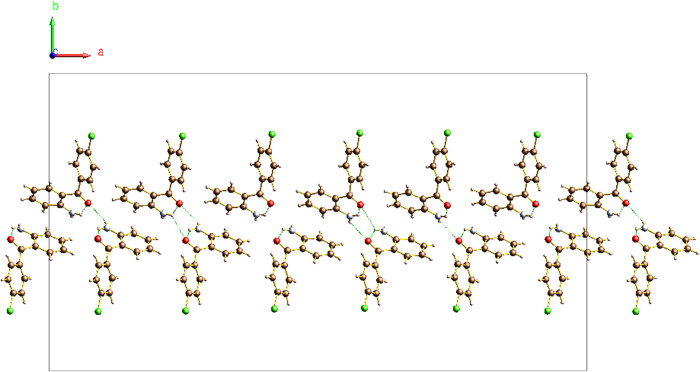
Ball and stick diagram of the 6-fold commensurate superstructure approximation of 2-amino-4′-chlorobenzophenone at 140 K, as viewed down the [001] direction. Only two columns of atoms depicting the variation of the hydrogen bonding network across the supercell are shown, for clarity. The hydrogen bonds are depicted by green dotted lines.

### Mechanism

The nature of the phase transition of 2-amino-4′-bromobenzophenone can be determined by considering why the structure is a nonmerohedral twin at 150 K and not a single crystal. The phase transition seems to conform neatly to the classical description of a second order phase transition in that it appears to be continuous and the result of a small distortion in the structure.^29^ In terms of the mechanism, considering the “nucleation and growth” model commonly applied to transitions in molecular crystals it can be inferred that in the case of 2-amino-4′-bromobenzophenone there are many nucleation sites throughout the crystal, and this is the root of the twinning.

If there were one nucleation site for the transition with growth proceeding in cooperative molecule-by-molecule fashion propagating throughout the crystal, one would expect that the transition would be truly single-crystal-to-single-crystal with only one orientation observed. As two twin domains are observed, logically there must be multiple nucleation events, and as all molecules in the orthorhombic structure are equivalent, any one in each pair of molecules along the screw axis in the [001] direction at 190 K could potentially change conformation at 150 K and hence, two distinct distortions of the original cell are possible.

That the two twin domains are dependent on which of two molecules along the screw axis change conformation, it makes sense that the twin law is a 2-fold rotation about [100] at 150 K. As each molecule has the same probability of being the one to change conformation, one would anticipate a 50:50 distribution of twin domains and this is exactly what is observed lending further credence to this interpretation.

In summary, as the crystal is cooled and the intermolecular distances decrease, this contraction creates strain on the structure up to the point where a rearrangement is necessary. At this point the phase transition is triggered at multiple points throughout the crystal and depending on the direction of the molecule that rearranges relative to the screw axis in the [001] direction at 190 K, one of two twin domains is formed with equal probability.

On the whole, the relationship between the phases is reminiscent of that between the high and low-temperature polymorphs of the citrate salt of diphenhydramine,^10^ which similarly transitions from an orthorhombic form (*P*2_1_2_1_2_1_) at high-temperature to a lower symmetry monoclinic form (*P*2_1_) at low-temperature with an accompanying nonmerohedral twinning. In this case, the transition itself is classed as first order proceeding in a more abrupt fashion with little in the way of hysteresis.

In contrast, as the crystal of 2-amino-4′-chlorobenzophenone is cooled and the intermolecular distances contract, this allows distinct competing hydrogen bonding networks between neighboring molecules to develop. The smaller atomic radius of chlorine in comparison to bromine is proposed to subtly influence the packing of the structure, which allows the N1 and O1 atoms to approach close enough to one another that multiple structurally related packing arrangements (stabilized by distinct hydrogen bonding networks) are energetically feasible.

### High-Pressure Measurements

Having investigated the behavior of crystals of both 2-amino-4′-halobenzophenones at different temperatures, the decision was made to look into the effect that compression by means of single crystal high-pressure X-ray diffraction experiments may have on these systems, since the origin of the modulation in 2-amino-4′-chlorobenzophenone was proposed to arise due to the evolution of short contacts between the atoms involved in hydrogen bond formation.

Upon compression of 2-amino-4′-chlorobenzophenone, a phase transition was observed between 0.16 and 0.66 GPa, which was characterized by a diffraction pattern that exhibited signs of incommensurate modulation (Figure 15). Although the crystal system remained unchanged (oP), a slight elongation of the *b*-axis was observed, from 25.009(13) Å at 0.16 GPa to 25.138(18) Å at 0.66 GPa (Supporting Information Figure S3). The refined modulation vector, *q*, was found to change as a function of pressure (Supporting Information Table S6), evolving from *q* = (0.1793(4), 0, 0) at 0.47 GPa to *q* = (0.2453(3), 0, 0) at 1.62 GPa. Upon decompression of the cell, further data points were collected that demonstrated the phase transition to be reversible, as the original phase was recovered upon decompression of the cell from 0.47 to 0.25 GPa, with minimal sample degradation observed (Supporting Information Table S9).

**Figure 15:**
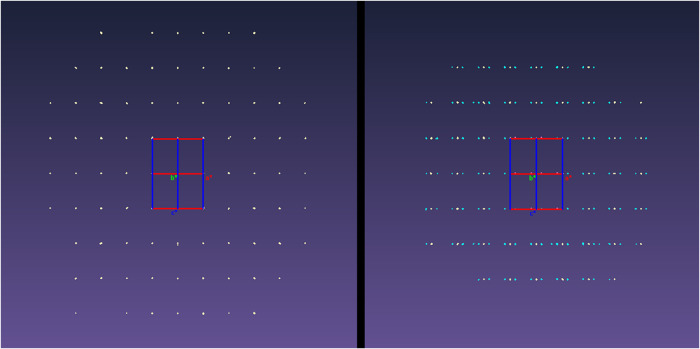
Reciprocal lattices of 2-amino-4′-chlorobenzophenone collected at 0.16 GPa (left) and 0.66 GPa (right), as viewed down the b* lattice vectors. The main reflections and satellite reflections are depicted in pale yellow and cyan, respectively.

Similarly, compression of 2-amino-4′-bromobenzophenone resulted in a phase transition between 0.36 and 0.69 GPa to a modulated phase (Figure 16). The diffraction pattern displayed satellite reflections similar to the high-pressure phase of 2-amino-4′-chlorobenzophenone and the phase transition was likewise accompanied by an increase in the *b*-axis length from 25.293(11) Å at 0.36 GPa to 25.354(12) Å at 0.69 GPa (Supporting Information Table S5). The modulation vector was also found to change as a function of pressure, evolving from *q* = (0.19497(12),0, 0) at 0.60 GPa to *q* = (0.22554(10), 0, 0) at 1.70 GPa (Supporting Information Table S7). Additionally, due to the stronger diffraction from this analogue inside the DAC, higher order harmonics (m = 2 and 3) were observed experimentally. Decompression of the cell from 0.60 to 0.08 GPa showed the phase transition to be reversible, with minimal degradation to the crystal observed (Supporting Information Table S8).

**Figure 16:**
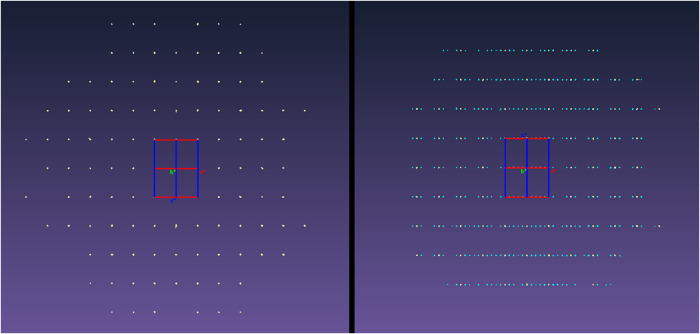
Reciprocal lattices of 2-amino-4′-bromobenzophenone collected at 0.36 GPa (left) and 0.69 GPa (right), as viewed down the b* lattice vectors. The main reflections and satellite reflections are depicted in pale yellow and cyan, respectively.

The high-pressure modulated data for 2-amino-4′-bromobenzophenone could be reduced with third-order harmonics accounted for (with the exception of the 1.70 GPa data set, where only the second-order harmonics were visible) to give reasonable R factors for both the main and satellite reflections, whereas only the first order harmonics were visible in the high-pressure data sets of 2-amino-4′-chlorobenzophenone (Supporting Information Tables S6 and S7). This is because the sample was too weakly diffracting for the higher-order harmonics to be observed experimentally inside the DAC, since the third-order harmonics of the 140 K collection at ambient pressure were clearly visible and could be treated effectively. However, as a result of the limited resolution, low data set completeness (∼30%), and in the case of 2-amino-4′-chlorobenzophenone, the weak diffraction of the sample when collected inside the DAC, solutions to the modulated structures of both compounds under elevated pressures proved elusive.

Average structures of the high-pressure phases were obtained by reducing the data using only the main reflections (thereby, ignoring the satellite reflections) and importing a reference model obtained at a lower pressure point. Because the average structures do not consider the modulation observed in the high-pressure phases, the resulting models do not accurately describe the motion or positions of the atoms within the crystals. Consequently, a full analysis and discussion of the differences between the ambient and high-pressure phases for both samples are precluded. However, given that the modulated diffraction patterns of both high-pressure modulated phases are strikingly similar to that observed for 2-amino-4′-chlorobenzophenone under ambient pressure at 140 K (both in relative intensity and position), this strongly suggests that the resulting structural modulation that occurs upon compression of both 2-amino-4′-bromobenzophenone and 2-amino-4′-chlorobenzophenone emulates the modulation observed in 2-amino-4′-chlorobenzophenone upon cooling to 140 K.

## Conclusions

It has been shown that between 150 and 290 K, 2-amino-4′-bromobenzophenone exists as one of two polymorphs with an enantiotropic second order phase transition occurring between 180 and 185 K when heated and 175–180 K when cooled. The transition to the lower temperature phase is accompanied by a reduction in symmetry (*Pna*2_1_ to *Pa*) in the structure and a nonmerohedral twinning manifest in the diffraction pattern. It can be inferred that this twinning is likely the result of a nucleation and growth mechanism with multiple nucleation points throughout the crystal.

Contrastingly, 2-amino-4′-chlorobenzophenone was found to undergo a reversible phase transition between 140 and 150 K when studied between the temperatures of between 290 and 140 K. The structures transforms from a periodic *Pna*2_1_ phase to an incommensurate phase in the superspace group *Pna*2_1_(α00)­000, with a refined q vector of (0.16255(11), 0, 0). A reasonable approximation of the true modulated structure can be described using a 6-fold commensurate supercell in the space group *Pn*. The origin of the modulation within 2-amino-4′-chlorobenzophenone has been attributed to the evolution of two distinct competing hydrogen-bonding networks as a result of the thermal contraction experienced by the structure upon cooling.

The hypothesis that the modulation observed arises due to the evolution of short contacts between atoms involved in the hydrogen bonding networks was supported by high-pressure X-ray diffraction experiments, which confirmed that compression of both 2-amino-4′-halobenzophenones resulted in the formation of high-pressure phases exhibiting incommensurate modulation. The characteristics of the modulation observed in the high-pressure phases of the 2-amino-4′-halobenzophenones emulate the modulation observed in 2-amino-4′-chlorobenzophenone at 140 K, suggesting that the structures at elevated pressures are similar the 140 K structure of 2-amino-4′-chlorobenzophenone.

This study provides valuable insights into a rare phase transition accompanied by the formation of a crystal twinning. As well as being an interesting crystallographic case study, this work is also important in the context of polymorphism. Given the significance of polymorphism to a variety of different scientific disciplines, a systematic study such as this in which a transformation from one polymorph to another can be visualized at the molecular level will provide insights that could lead to more efficient control and design of polymorphic forms.

In addition, this study demonstrates the importance of revisiting crystal structures for which only room temperature data are available. There are likely many more interesting phase transitions yet to be discovered.

## Supplementary Material



## Notes

Full structure refinement details for both variable temperature and variable pressure experiments in addition to complementary temperature and pressure profile graphs and unwarp frames showing the modulation in both 2-amino-4′-halobenzophenones (PDF)
