# Supplementary material for: Low-Temperature and High-Pressure Phase Transitions in Two 2‑Amino-4′-halobenzophenones: Incommensurate Modulation and a Case of Temperature-Induced Twinning
Source: Cryst Growth Des. 2026 Feb 6;26(4):1715–28. doi: 10.1021/acs.cgd.5c01520 (PMC12921689; doi:10.1021/acs.cgd.5c01520)
Supplement: Supplementary file 1 [file cg5c01520_si_001.pdf]

Supplementary Information

Low-temperature and high-pressure phase transitions in two 2-Amino-4’-halobenzophenones: incommensurate modulation and a case of temperature-induced twinning

Lani Attiwell[1], Max T. Hill[2,3], Jonathan D. Sellars[2,3], Lukáš Palatinus[4], Alexandra Longcake[1]*, Paul G. Waddell[1]*

[1]School of Natural and Environmental Sciences, Bedson Building, Newcastle University, Newcastle upon Tyne, NE1 7RU, UK
[2] Biosciences Institute, Faculty of Medical Sciences, Newcastle University, Newcastle upon Tyne, NE1 7RU, UK.
[3] School of Pharmacy, Faculty of Medical Sciences, Newcastle University, Newcastle upon Tyne, NE1 7RU, UK.
[4]Department of Structure Analysis, Institute of Physics of the Czech Academy of Sciences, Na Slovance 1999/2, Prague 8, Czechia.

Contents

2-amino-4’-bromobenzophenone variable temperature data:..2
2-amino-4’-chlorobenzophenone variable temperature data: 4
2-amino-4’-bromobenzophenone high pressure data: 7
2-amino-4’-chlorobenzophenone high pressure data:..12

2-amino-4'-bromobenzophenone variable temperature data:

Table S1 Crystal data and structural refinement details for 2-amino-4'-bromobenzophenone

CCDC Number	2497476	2497475
Temperature	150 K (second collection)	290 K
Empirical formula	$C_{13}H_{10}BrNO$	
Formula weight	276.13	
Crystal system	monoclinic	orthorhombic
Space group	*Pa*	*Pna*2_1
a/Å	7.67657(12)	7.8159(3)
b/Å	25.5846(4)	25.4029(9)
c/Å	5.76556(8)	5.8473(2)
α/°	90	90
β/°	92.6274(14)	90
γ/°	90	90
Volume/Å^3	1131.18(3)	1160.96(7)
Z	4	4
ρ_{calc}g/cm^3	1.621	1.580
μ/mm^{-1}	4.757	4.635
F(000)	552.0	552.0
Crystal size/mm^3	0.19 × 0.07 × 0.02	0.18 × 0.07 × 0.03
Radiation	CuKα (λ = 1.54184)	CuKα (λ = 1.54184)
2Θ range for data collection/°	6.91 to 146.876	6.96 to 146.596
Index ranges	-9 ≤ h ≤ 9, -31 ≤ k ≤ 31, -7 ≤ l ≤ 6	-9 ≤ h ≤ 9, -29 ≤ k ≤ 30, -4 ≤ l ≤ 6
Reflections collected	18130	4746
Independent reflections	3416 [R_{int} = 0.0259, R_{sigma} = 0.0192]	1688 [R_{int} = 0.0159, R_{sigma} = 0.0182]
Data/restraints/parameters	18130/2/302	1688/3/151
Goodness-of-fit on F^2	1.043	1.067
Final R indexes [I>=2σ (I)]	R_1 = 0.0249, wR_2 = 0.0675	R_1 = 0.0235, wR_2 = 0.0668
Final R indexes [all data]	R_1 = 0.0255, wR_2 = 0.0680	R_1 = 0.0242, wR_2 = 0.0674
Largest diff. peak/hole / e Å^{-3}	0.32/-0.28	0.18/-0.34
Flack parameter	-0.014(11)	-0.052(19)

Table S2 Unit cell parameters for 2-amino-4'-bromobenzophenone at various temperatures. Measurements from full datasets are rendered in bold.

Temperature/K	a /Å	b /Å	c /Å	β/°
**150**	**7.67458(10)**	**25.5980(3)**	**5.76382(8)**	**92.6639(11)**
155	7.670(4)	25.602(12)	5.817(10)	92.48(15)
160	7.694(5)	25.611(15)	5.818(13)	92.36(16)
165	7.698(4)	25.625(11)	5.822(10)	92.21(14)
170	7.700(4)	25.629(12)	5.816(11)	92.12(15)
175	7.718(4)	25.633(12)	5.811(10)	91.94(14)
180	7.726(6)	25.722(19)	5.755(12)	90.34(18)
185	7.756(3)	25.438(12)	5.785(3)	90
**190**	**7.7727(2)**	**25.4003(8)**	**5.7980(2)**	**90**
185	7.767(3)	25.441(9)	5.7888(13)	90
180	7.714(4)	25.532(12)	5.796(11)	90
175	7.696(4)	25.540(12)	5.786(10)	91.07(15)
170	7.680(6)	25.500(18)	5.770(16)	91.4(2)
165	7.708(4)	25.611(11)	5.763(9)	91.86(13)
160	7.690(5)	25.627(14)	5.819(15)	91.98(18)
155	7.674(4)	25.605(12)	5.809(10)	92.33(15)
**150**	**7.67657(12)**	**25.5846(4)**	**5.76556(8)**	**92.6274(14)**

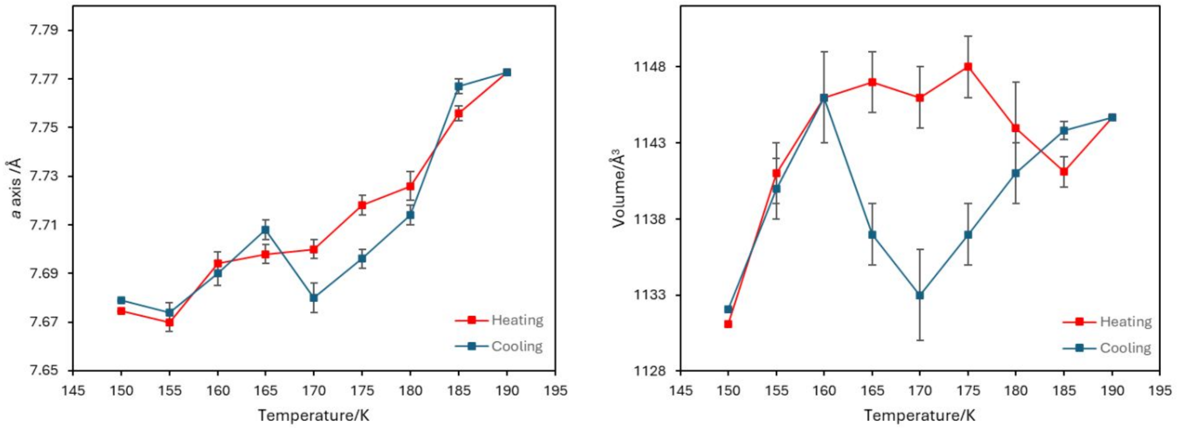

Fig. S1 The temperature profiles of the a-axis and unit cell volume 2-amino-4'-bromobenzophenone during heating and cooling.

2-amino-4'-chlorobenzophenone variable temperature data:

Table S3 Crystal data and structural refinement details for 2-amino-4'-chlorobenzophenone at 290 K.

CCDC Number	2497479
Temperature	290 K
Empirical formula	$C_{13}H_{10}ClNO$
Formula weight	231.67
Crystal system	orthorhombic
Space group	$Pna2_1$
a/Å	7.8314(2)
b/Å	25.1377(5)
c/Å	5.71420(10)
α/°	90
β/°	90
γ/°	90
Volume/Å^3	1124.92(4)
Z	4
ρ_{calc}g/cm^3	1.368
μ/mm^{-1}	2.806
F(000)	480.0
Crystal size/mm^3	0.18 × 0.07 × 0.02
Radiation	CuKα (λ = 1.54184)
2Θ range for data collection/°	7.032 to 146.134
Index ranges	-9 ≤ h ≤ 9, -31 ≤ k ≤ 30, -4 ≤ l ≤ 7
Reflections collected	9977
Independent reflections	1685 [R_{int} = 0.0213, R_{sigma} = 0.0138]
Data/restraints/parameters	1685/1/151
Goodness-of-fit on F^2	1.051
Final R indexes [I>=2σ (I)]	R_1 = 0.0271, wR_2 = 0.0764
Final R indexes [all data]	R_1 = 0.0286, wR_2 = 0.0780
Largest diff. peak/hole / e Å^{-3}	0.09/-0.16
Flack parameter	-0.028(14)

Table S4 Unit cell parameters for 2-amino-4'-chlorobenzophenone at various temperatures. Measurements from full datasets are rendered in bold.

Temperature/K	a /Å	b /Å	c /Å
**290**	**7.8314(2)**	**25.1377(5)**	**5.7142(1)**
280	7.831(4)	25.102(12)	5.7126(16)
270	7.824(4)	25.087(12)	5.7093(15)
260	7.828(4)	25.092(12)	5.7050(16)
250	7.821(4)	25.092(11)	5.6963(15)
240	7.823(3)	25.060(11)	5.6947(12)
230	7.815(3)	25.063(11)	5.6870(14)
220	7.817(3)	25.050(11)	5.6829(14)
210	7.805(3)	25.044(10)	5.6777(15)
200	7.811(3)	25.046(10)	5.6696(14)
**190**	**7.81582(16)**	**25.0555(6)**	**5.67004(13)**
180	7.813(3)	25.026(8)	5.6594(13)
170	7.813(3)	25.027(8)	5.6565(14)
160	7.813(3)	25.009(10)	5.6525(15)
150	7.836(6)	25.04(3)	5.648(3)
**140**	**7.6489(4)**	**25.2922(14)**	**5.6733(4)**

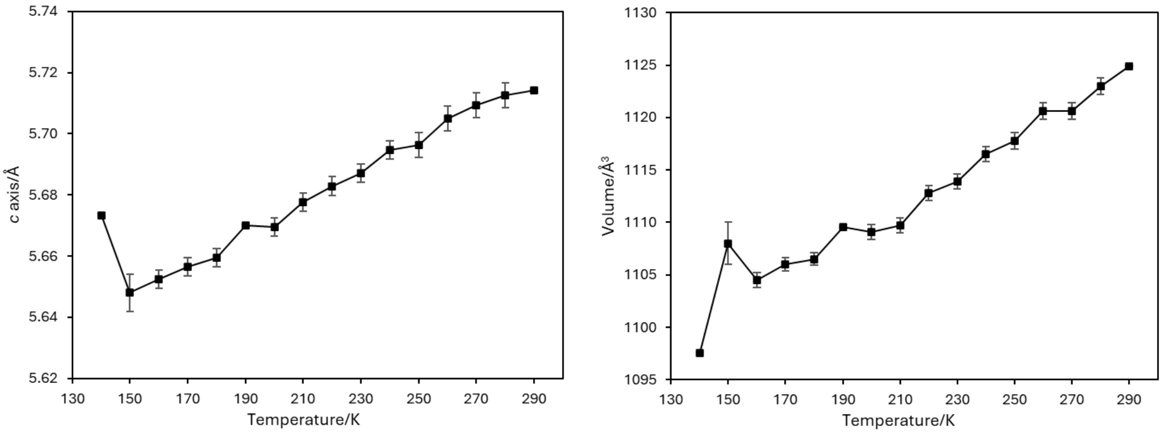

Fig. S2 The temperature profiles of the c-axis and unit cell volume 2-amino-4'-chlorobenzophenone during cooling.

Table S5 Crystal data and structural refinement details for the modulated structure of 2-amino-4'-chlorobenzophenone at 140 K.

CCDC number	2497498
Temperature	140 K
Empirical formula	$C_{13}H_{10}ClNO$
Formula weight	231.67
Crystal system	orthorhombic
Superspace group	$Pna2_1(\alpha00)000$
Modulation vector, q	0.16255(11), 0, 0
a/Å	7.6337(11)
b/Å	25.315(3)
c/Å	5.6567(11)
α/°	90
β/°	90
γ/°	90
Volume/$Å^3$	1093.1(3)
Z	4
$\rho_{calc}g/cm^3$	1.4077
μ/mm^{-1}	2.888
F(000)	480.0
Crystal size/mm^3	0.17 × 0.08 × 0.02
Radiation	CuKα (λ = 1.54184)
2Θ range for data collection/°	3.96 to 146.84
Index ranges	$-9 \leq h \leq 9$, $-31 \leq k \leq 30$, $-4 \leq l \leq 7$, $-3 \leq m_1 \leq 3$
Reflections collected	66777
Independent reflections	10933 [R_{int} = 0.0308, R_{sigma} = 0.0211]
Data/restraints/parameters	10933/0/1009
Goodness-of-fit on F^2	2.194
Final R indexes [I>=2σ (I)]	R_1 = 0.0347, wR_2 = 0.0850
Final R indexes [all data]	R_1 = 0.0505, wR_2 = 0.0897
Largest diff. peak/hole / e $Å^{-3}$	0.25/-0.34
Flack parameter	0.040(13)

2-amino-4'-bromobenzophenone high pressure data:

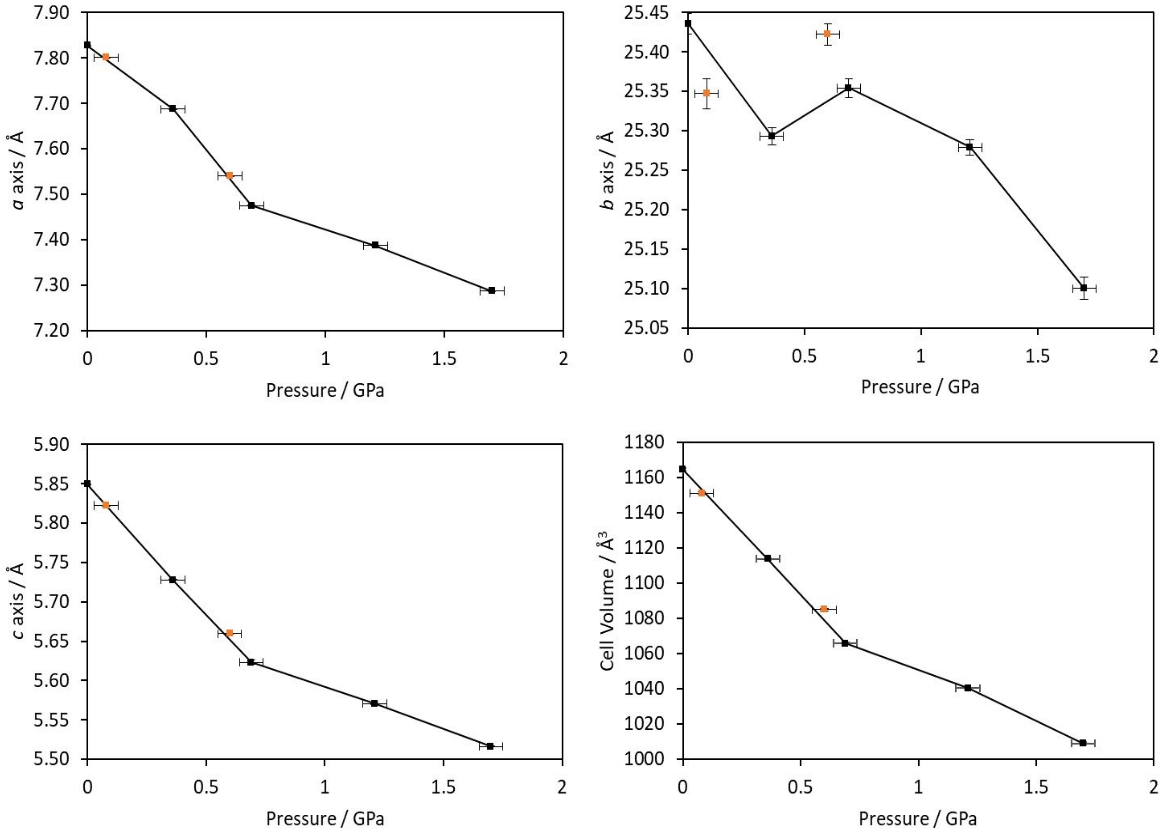

Figure S3 Graphs of the unit cell parameters for 2-amino-4'-bromobenzophenone as a function of pressure. Datapoints collected during compression and decompression are depicted in black and orange, respectively. Pressure error bars are supplied on figures as 0.5 times the magnitude of the individual pressure drift plus 0.05 GPa, attributed to the inherent uncertainty in the pressure determination method. Error bars associated with cell parameters are supplied on figures as the estimated standard deviation (esd) as calculated by SHELXL.

Table S6 Table of the modulation vector as a function of pressure for 2-amino-4'-bromobenzophenone, obtained after data reduction. Collections obtained upon decompression are denoted with an asterisk.

Pressure / GPa	Modulation vector, q	Harmonic order, m
**0.60***	0.19497(12), -0.00015(18) , 0.00012(8)	1
**0.69**	0.19796(8), 0, 0	3
**1.21**	0.20456(11), 0.0012(2), 0	3
**1.70**	0.22554(10), -0.00022(16), 0	2

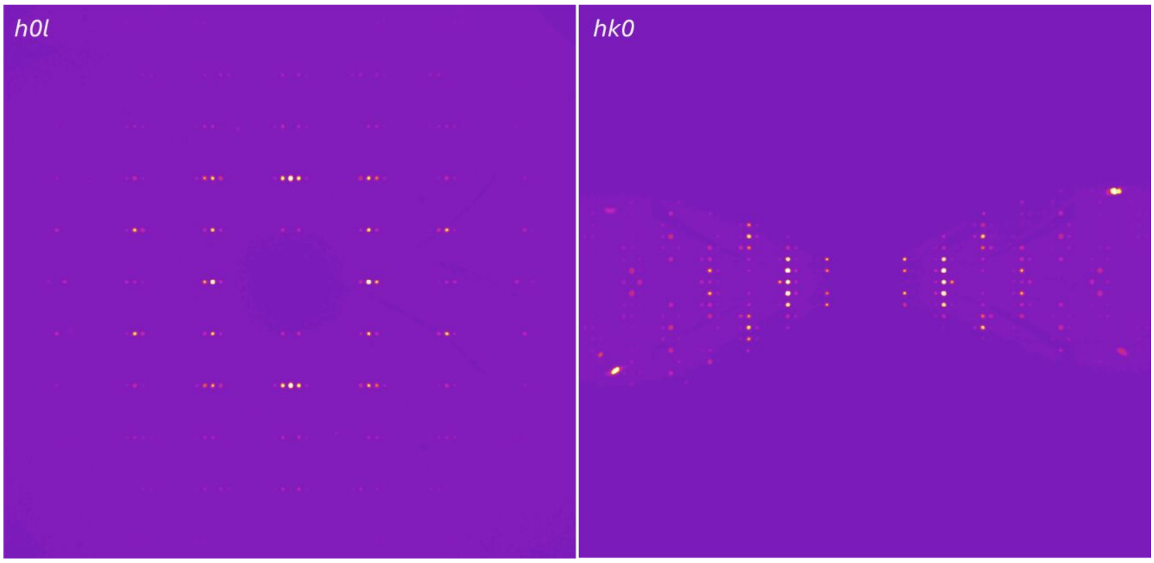

Figure S4 Selected unwarp frames from the collection of 2-amino-4’-bromobenzophenone at 1.21 GPa, depicting the modulation evident in the *h* plane.

Table S7 Crystal data and structural refinement details for the high-pressure datasets of 2-amino-4'-bromobenzophenone.

CCDC number	2497481	2497482	2497483
Pressure	Ambient pressure	0.0 GPa	0.36 GPa
Empirical formula	$C_{13}H_{10}NOBr$	$C_{13}H_{10}NOBr$	$C_{13}H_{10}NOBr$
Formula weight	276.13	276.13	276.13
Temperature/K	293(2)	293(2)	293(2)
Crystal system	orthorhombic	orthorhombic	orthorhombic
Space group	$Pna2_1$	$Pna2_1$	$Pna2_1$
a/Å	7.82580(10)	7.8275(3)	7.6874(3)
b/Å	25.4087(3)	25.436(13)	25.293(11)
c/Å	5.84810(10)	5.8494(2)	5.72802(18)
α/°	90	90	90
β/°	90	90	90
γ/°	90	90	90
Volume/$Å^3$	1162.86(3)	1164.6(6)	1113.8(5)
Z	4	4	4
$\rho_{calc}g/cm^3$	1.577	1.575	1.647
μ/mm^{-1}	4.627	3.506	3.666
F(000)	552.0	552.0	552.0
Crystal size/mm^3	0.182 × 0.104 × 0.066	0.182 × 0.104 × 0.066	0.182 × 0.104 × 0.066
Radiation	Cu Kα (λ = 1.54184)	Mo Kα (λ = 0.71073)	Mo Kα (λ = 0.71073)
2Θ range for data collection/°	6.958 to 152.396	5.446 to 46.576	5.538 to 46.42
Index ranges	-9 ≤ h ≤ 9, -30 ≤ k ≤ 31, -7 ≤ l ≤ 6	-8 ≤ h ≤ 8, -7 ≤ k ≤ 7, -6 ≤ l ≤ 6	-8 ≤ h ≤ 8, -7 ≤ k ≤ 7, -6 ≤ l ≤ 6
Reflections collected	20987	11334	10002
Independent reflections	2340 [R_{int} = 0.0260, R_{sigma} = 0.0118]	590 [R_{int} = 0.0291, R_{sigma} = 0.0120]	571 [R_{int} = 0.0272, R_{sigma} = 0.0112]
Data/restraints/parameters	2340/120/151	590/52/70	571/52/70
Goodness-of-fit on F^2	1.044	1.076	1.069
Final R indexes [I>=2σ (I)]	R_1 = 0.0203, wR_2 = 0.0568	R_1 = 0.0430, wR_2 = 0.1002	R_1 = 0.0455, wR_2 = 0.1178
Final R indexes [all data]	R_1 = 0.0208, wR_2 = 0.0572	R_1 = 0.0460, wR_2 = 0.1027	R_1 = 0.0514, wR_2 = 0.1238
Largest diff. peak/hole / e $Å^{-3}$	0.19/-0.29	0.22/-0.17	0.24/-0.18
Flack parameter	-0.018(8)	0.009(9)	-0.014(10)

CCDC number	2497484	2497485	2497486
Pressure	0.69 GPa*	1.21 GPa*	1.70 GPa*
Empirical formula	$C_{13}H_{10}NOBr$	$C_{13}H_{10}NOBr$	$C_{13}H_{10}NOBr$
Formula weight	276.13	276.13	276.13
Temperature/K	293(2)	293(2)	293(2)
Crystal system	orthorhombic	orthorhombic	orthorhombic
Space group	$Pna2_1$	$Pna2_1$	$Pna2_1$
a/Å	7.4761(2)	7.38908(20)	7.2872(3)
b/Å	25.344(12)	25.279(10)	25.100(14)
c/Å	5.6236(3)	5.5727(2)	5.5163(3)
α/°	90	90	90
β/°	90	90	90
γ/°	90	90	90
Volume/$Å^3$	1065.5(5)	1040.9(4)	1009.0(6)
Z	4	4	4
$\rho_{calc}g/cm^3$	1.721	1.762	1.818
μ/mm^{-1}	3.832	3.923	4.047
F(000)	552.0	552.0	552.0
Crystal size/mm^3	0.182 × 0.104 × 0.066	0.182 × 0.104 × 0.066	0.182 × 0.104 × 0.066
Radiation	Mo Kα (λ = 0.71073)	Mo Kα (λ = 0.71073)	Mo Kα (λ = 0.71073)
2Θ range for data collection/°	5.682 to 43.8	5.744 to 41.592	5.822 to 41.642
Index ranges	-7 ≤ h ≤ 7, -7 ≤ k ≤ 7, -5 ≤ l ≤ 5	-7 ≤ h ≤ 7, -7 ≤ k ≤ 7, -5 ≤ l ≤ 5	-7 ≤ h ≤ 7, -7 ≤ k ≤ 7, -5 ≤ l ≤ 5
Reflections collected	9495	9158	8250
Independent reflections	461 [R_{int} = 0.0272, R_{sigma} = 0.0114]	418 [R_{int} = 0.0276, R_{sigma} = 0.0100]	403 [R_{int} = 0.0321, R_{sigma} = 0.0107]
Data/restraints/parameters	461/55/70	418/56/70	403/58/70
Goodness-of-fit on F^2	1.107	1.090	1.068
Final R indexes [I>=2σ (I)]	R_1 = 0.0817, wR_2 = 0.1579	R_1 = 0.0830, wR_2 = 0.1780	R_1 = 0.0844, wR_2 = 0.1685
Final R indexes [all data]	R_1 = 0.0898, wR_2 = 0.1637	R_1 = 0.0908, wR_2 = 0.1843	R_1 = 0.0922, wR_2 = 0.1736
Largest diff. peak/hole / e $Å^{-3}$	0.25/-0.29	0.25/-0.30	0.28/-0.28
Flack parameter	0.10(3)	0.11(5)	0.12(5)

*Average structure at this pressure

CCDC number	2497487	2497488
Pressure	0.60 GPa*	0.08 GPa
Empirical formula	$C_{13}H_{10}NOBr$	$C_{13}H_{10}NOBr$
Formula weight	276.13	276.13
Temperature/K	293(2)	293(2)
Crystal system	orthorhombic	orthorhombic
Space group	$Pna2_1$	$Pna2_1$
a/Å	7.5407(3)	7.8006(4)
b/Å	25.422(14)	25.347(19)
c/Å	5.6595(3)	5.8223(3)
α/°	90	90
β/°	90	90
γ/°	90	90
Volume/Å^3	1084.9(6)	1151.2(9)
Z	4	4
ρ_{calc}g/cm^3	1.691	1.593
μ/mm^{-1}	3.764	3.547
F(000)	552.0	552.0
Crystal size/mm^3	0.182 × 0.104 × 0.066	0.182 × 0.104 × 0.066
Radiation	Mo Kα (λ = 0.71073)	Mo Kα (λ = 0.71073)
2Θ range for data collection/°	5.636 to 43.778	5.464 to 43.968
Index ranges	-7 ≤ h ≤ 7, -7 ≤ k ≤ 7, -5 ≤ l ≤ 5	-8 ≤ h ≤ 8, -7 ≤ k ≤ 7, -6 ≤ l ≤ 6
Reflections collected	9174	9954
Independent reflections	464 [R_{int} = 0.0454, R_{sigma} = 0.0166]	515 [R_{int} = 0.0582, R_{sigma} = 0.0192]
Data/restraints/parameters	464/67/70	515/54/70
Goodness-of-fit on F^2	1.292	1.315
Final R indexes [I>=2σ (I)]	R_1 = 0.1308, wR_2 = 0.2345	R_1 = 0.0826, wR_2 = 0.1567
Final R indexes [all data]	R_1 = 0.1312, wR_2 = 0.2346	R_1 = 0.0834, wR_2 = 0.1570
Largest diff. peak/hole / e Å^{-3}	0.45/-0.42	0.38/-0.31
Flack parameter	0.08(5)	0.004(15)

*Average structure at this pressure

2-amino-4'-chlorobenzophenone high pressure data:

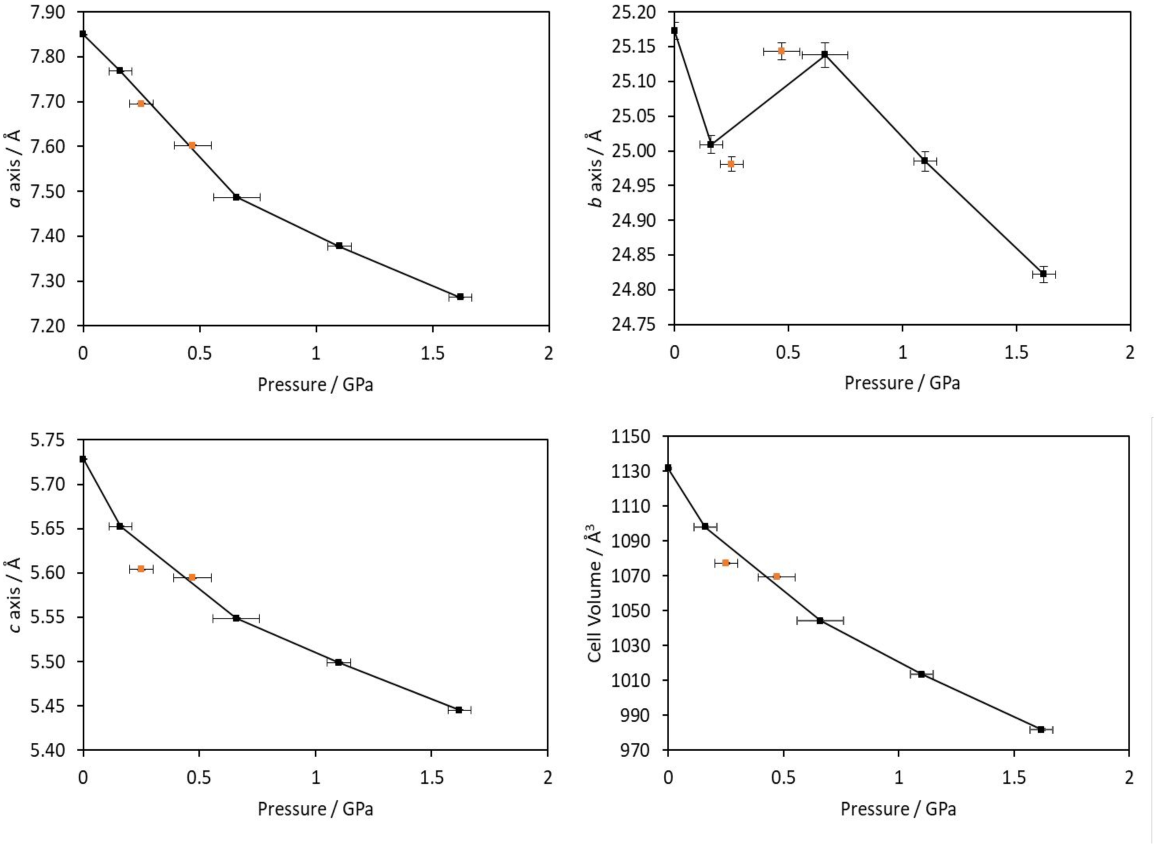

Figure S5 Graphs of the unit cell parameters for 2-amino-4'-chlorobenzophenone as a function of pressure. Datapoints collected during compression and decompression are depicted in black and orange, respectively. Pressure error bars are supplied on figures as 0.5 times the magnitude of the individual pressure drift plus 0.05 GPa, attributed to the inherent uncertainty in the pressure determination method. Error bars associated with cell parameters are supplied on figures as the estimated standard deviation (esd) as calculated by SHELXL.

Table S8 Table of the modulation vector as a function of pressure for 2-amino-4'-chlorobenzophenone, obtained after data reduction. Collections obtained upon decompression are denoted with an asterisk.

Pressure / GPa	Modulation vector, q	Satellite order, m
**0.47***	0.1793(4), 0, 0	1
**0.66**	0.2015(4), 0, 0	1
**1.10**	0.2218(3), 0, 0	1
**1.62**	0.2453(3), 0, 0	1

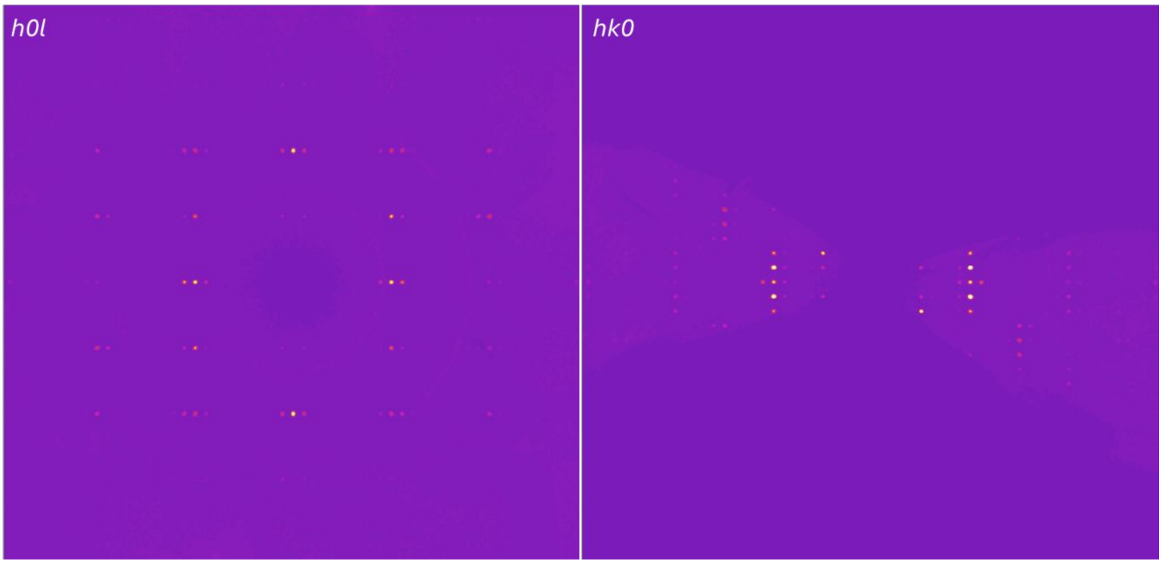

Figure S6 Selected unwarp frames from the collection of 2-amino-4'-chlorobenzophenone at 1.10 GPa, depicting the modulation evident in the *h* plane.

Table S9 Crystal data and structural refinement details for the high pressure datasets of 2-amino-4'-chlorobenzophenenone

CCDC number	2497489	2497490	2497491
Pressure	Ambient pressure	0.0 GPa	0.16 GPa
Empirical formula	$C_{13}H_{10}ClNO$	$C_{13}H_{10}ClNO$	$C_{13}H_{10}ClNO$
Formula weight	231.67	231.67	231.67
Temperature/K	293(2)	293(2)	293(2)
Crystal system	orthorhombic	orthorhombic	orthorhombic
Space group	$Pna2_1$	$Pna2_1$	$Pna2_1$
a/Å	7.83840(10)	7.8501(6)	7.7680(6)
b/Å	25.1582(2)	25.173(12)	25.009(13)
c/Å	5.72070(10)	5.7280(4)	5.6524(4)
α/°	90	90	90
β/°	90	90	90
γ/°	90	90	90
Volume/Å^3	1128.12(3)	1131.9(5)	1098.1(6)
Z	4	4	4
ρ_{calc}g/cm^3	1.364	1.359	1.401
μ/mm^{-1}	2.798	0.313	0.323
F(000)	480.0	480.0	480.0
Crystal size/mm^3	0.205 × 0.049 × 0.036	0.205 × 0.049 × 0.036	0.205 × 0.049 × 0.036
Radiation	Cu Kα (λ = 1.54184)	Mo Kα (λ = 0.71073)	Mo Kα (λ = 0.71073)
2Θ range for data collection/°	7.028 to 152.484	5.436 to 34.446	5.492 to 37.674
Index ranges	-9 ≤ h ≤ 9, -31 ≤ k ≤ 31, -5 ≤ l ≤ 6	-6 ≤ h ≤ 6, -10 ≤ k ≤ 10, -4 ≤ l ≤ 4	-7 ≤ h ≤ 7, -10 ≤ k ≤ 10, -5 ≤ l ≤ 5
Reflections collected	27393	4945	5827
Independent reflections	2132 [R_{int} = 0.0218, R_{sigma} = 0.0092]	372 [R_{int} = 0.0464, R_{sigma} = 0.0218]	435 [R_{int} = 0.0491, R_{sigma} = 0.0257]
Data/restraints/parameters	2132/120/151	372/53/70	435/53/70
Goodness-of-fit on F^2	1.040	1.082	1.187
Final R indexes [I>=2σ (I)]	R_1 = 0.0265, wR_2 = 0.0753	R_1 = 0.0582, wR_2 = 0.1194	R_1 = 0.0591, wR_2 = 0.1193
Final R indexes [all data]	R_1 = 0.0273, wR_2 = 0.0762	R_1 = 0.0664, wR_2 = 0.1262	R_1 = 0.0675, wR_2 = 0.1249
Largest diff. peak/hole / e Å^{-3}	0.13/-0.15	0.22/-0.14	0.22/-0.15
Flack parameter	-0.007(5)	-0.01(7)	0.12(7)

CCDC number	2497492	2497493	2497494
Pressure	0.66 GPa*	1.10 GPa*	1.62 GPa*
Empirical formula	$C_{13}H_{10}ClNO$	$C_{13}H_{10}ClNO$	$C_{13}H_{10}ClNO$
Formula weight	231.67	231.67	231.67
Temperature/K	293(2)	293(2)	293(2)
Crystal system	orthorhombic	orthorhombic	orthorhombic
Space group	$Pna2_1$	$Pna2_1$	$Pna2_1$
a/Å	7.4871(7)	7.3751(6)	7.2602(4)
b/Å	25.133(18)	24.983(15)	24.835(12)
c/Å	5.5485(6)	5.4976(5)	5.4416(4)
α/°	90	90	90
β/°	90	90	90
γ/°	90	90	90
Volume/Å^3	1044.1(8)	1012.9(6)	981.1(5)
Z	4	4	4
ρ_{calc}g/cm^3	1.474	1.519	1.568
μ/mm^{-1}	0.339	0.350	0.361
F(000)	480.0	480.0	480.0
Crystal size/mm^3	0.205 × 0.049 × 0.036	0.205 × 0.049 × 0.036	0.205 × 0.049 × 0.036
Radiation	Mo Kα (λ = 0.71073)	Mo Kα (λ = 0.71073)	Mo Kα (λ = 0.71073)
2Θ range for data collection/°	5.678 to 30.484	5.76 to 32.82	5.846 to 30.522
Index ranges	-5 ≤ h ≤ 5, -9 ≤ k ≤ 9, -4 ≤ l ≤ 4	-5 ≤ h ≤ 5, -10 ≤ k ≤ 10, -4 ≤ l ≤ 4	-5 ≤ h ≤ 5, -9 ≤ k ≤ 9, -4 ≤ l ≤ 4
Reflections collected	2613	3266	3318
Independent reflections	237 [R_{int} = 0.0753, R_{sigma} = 0.0327]	297 [R_{int} = 0.0453, R_{sigma} = 0.0220]	227 [R_{int} = 0.0468, R_{sigma} = 0.0182]
Data/restraints/parameters	237/57/65	297/59/65	227/58/65
Goodness-of-fit on F^2	1.216	1.130	1.285
Final R indexes [I>=2σ (I)]	R_1 = 0.1257, wR_2 = 0.3582	R_1 = 0.1482, wR_2 = 0.4165	R_1 = 0.1398, wR_2 = 0.3747
Final R indexes [all data]	R_1 = 0.1301, wR_2 = 0.3751	R_1 = 0.1579, wR_2 = 0.4319	R_1 = 0.1457, wR_2 = 0.3938
Largest diff. peak/hole / e Å^{-3}	0.26/-0.32	0.39/-0.44	0.28/-0.46
Flack parameter	0.15(14)	0.05(8)	-0.02(6)

*Average structure at this pressure

CCDC number	2497495	2497496
Pressure	0.47 GPa*	0.25 GPa
Empirical formula	$C_{13}H_{10}ClNO$	$C_{13}H_{10}ClNO$
Formula weight	231.67	231.67
Temperature/K	293(2)	293(2)
Crystal system	orthorhombic	orthorhombic
Space group	$Pna2_1$	$Pna2_1$
a/Å	7.6038(5)	7.6952(5)
b/Å	25.123(12)	24.981(10)
c/Å	5.5963(4)	5.6041(3)
α/°	90	90
β/°	90	90
γ/°	90	90
Volume/Å^3	1069.1(5)	1077.3(5)
Z	4	4
ρ_{calc}g/cm^3	1.439	1.428
μ/mm^{-1}	0.331	0.329
F(000)	480.0	480.0
Crystal size/mm^3	0.205 × 0.049 × 0.036	0.205 × 0.049 × 0.036
Radiation	Mo Kα (λ = 0.71073)	Mo Kα (λ = 0.71073)
2Θ range for data collection/°	5.598 to 31.666	5.54 to 37.536
Index ranges	-5 ≤ h ≤ 5, -9 ≤ k ≤ 9, -4 ≤ l ≤ 4	-6 ≤ h ≤ 6, -10 ≤ k ≤ 10, -5 ≤ l ≤ 5
Reflections collected	4084	5824
Independent reflections	276 [R_{int} = 0.0396, R_{sigma} = 0.0177]	417 [R_{int} = 0.0403, R_{sigma} = 0.0210]
Data/restraints/parameters	276/57/65	417/54/70
Goodness-of-fit on F^2	1.122	1.104
Final R indexes [I>=2σ (I)]	R_1 = 0.0900, wR_2 = 0.2440	R_1 = 0.0630, wR_2 = 0.1107
Final R indexes [all data]	R_1 = 0.0975, wR_2 = 0.2542	R_1 = 0.0718, wR_2 = 0.1176
Largest diff. peak/hole / e Å^{-3}	0.22/-0.22	0.22/-0.18
Flack parameter	0.02(8)	0.09(6)

*Average structure at this pressure
